# Characterization of the Antigenic Heterogeneity of Lipoarabinomannan, the Major Surface Glycolipid of *Mycobacterium tuberculosis*, and Complexity of Antibody Specificities toward This Antigen

**DOI:** 10.4049/jimmunol.1701673

**Published:** 2018-04-02

**Authors:** Alok Choudhary, Deendayal Patel, William Honnen, Zhong Lai, Raja Sekhar Prattipati, Ruixiang Blake Zheng, Ying-Chao Hsueh, Maria Laura Gennaro, Alfred Lardizabal, Blanca I. Restrepo, Moncerrato Garcia-Viveros, Maju Joe, Yu Bai, Ke Shen, Kamar Sahloul, John S. Spencer, Delphi Chatterjee, Tobias Broger, Todd L. Lowary, Abraham Pinter

**Affiliations:** * Public Health Research Institute, New Jersey Medical School, Rutgers, The State University of New Jersey, Newark, NJ 07103;; † Alberta Glycomics Centre and Department of Chemistry, University of Alberta, Edmonton, Alberta T6G 2G2, Canada;; ‡ Global Tuberculosis Institute, New Jersey Medical School, Rutgers, The State University of New Jersey, Newark, NJ 07103;; § University of Texas Health Science Center at Houston, School of Public Health at Brownsville, Brownsville, TX 78520;; ¶ Secretaria de Salud de Tamaulipas, Ciudad Victoria 87000, Mexico;; ‖ Mycobacteria Research Laboratories, Department of Microbiology, Immunology, and Pathology, Colorado State University, Fort Collins, CO 80523; and; # Foundation for Innovative New Diagnostics, Geneva 1202, Switzerland

## Abstract

Lipoarabinomannan (LAM), the major antigenic glycolipid of *Mycobacterium tuberculosis*, is an important immunodiagnostic target for detecting tuberculosis (TB) infection in HIV-1–coinfected patients, and is believed to mediate a number of functions that promote infection and disease development. To probe the human humoral response against LAM during TB infection, several novel LAM-specific human mAbs were molecularly cloned from memory B cells isolated from infected patients and grown in vitro. The fine epitope specificities of these Abs, along with those of a panel of previously described murine and phage-derived LAM-specific mAbs, were mapped using binding assays against LAM Ags from several mycobacterial species and a panel of synthetic glycans and glycoconjugates that represented diverse carbohydrate structures present in LAM. Multiple reactivity patterns were seen that differed in their specificity for LAM from different species, as well as in their dependence on arabinofuranoside branching and nature of capping at the nonreducing termini. Competition studies with mAbs and soluble glycans further defined these epitope specificities and guided the design of highly sensitive immunodetection assays capable of detecting LAM in urine of TB patients, even in the absence of HIV-1 coinfection. These results highlighted the complexity of the antigenic structure of LAM and the diversity of the natural Ab response against this target. The information and novel reagents described in this study will allow further optimization of diagnostic assays for LAM and may facilitate the development of potential immunotherapeutic approaches to inhibit the functional activities of specific structural motifs in LAM.

## Introduction

Tuberculosis (TB) remains one of the world’s deadliest communicable diseases, with approximately one third of the world’s population currently infected with *Mycobacterium tuberculosis*, and an estimated 10.4 million new cases of TB disease and 1.7 million deaths from the disease in 2015 (1). Although there is a long-established paradigm that immunity against *M. tuberculosis* relies primarily on cellular defense mechanisms, this paradigm has been questioned by increasing evidence for the importance of Ab-mediated immunity (2–9), with recent studies suggesting that the isotypes of TB-specific Abs (10) and the glycan composition at positions in the Fc domain (11) contributed to the level of protection. Despite this renewed emphasis, our understanding of the contribution of the humoral immune response toward controlling *M. tuberculosis* has been hindered by the limited characterizations of the humoral response to *M. tuberculosis* infection, particularly in the human host.

The *M. tuberculosis* surface glycolipid, lipoarabinomannan (LAM), is a prominent humoral target. LAM is a major structural component of the *M. tuberculosis* cell wall and an important mediator of functions that promote productive infection and pathogenicity (reviewed in Refs. 12, 13). LAM contains four distinct structural domains: a phosphatidylinositol anchor, an α-(1→6)–linked mannan backbone of mannopyranose (Man*p*) residues with pendant α-(1→2)–Man*p*-linked side chains, an arabinan chain containing multiple arabinofuranoside (Ara*f*) residues with tetra- and hexa-Ara*f* termini, and terminal caps containing various carbohydrate motifs. Fast-growing nonpathogenic strains, such as *Mycobacterium smegmatis*, have uncapped ends or inositol phosphate caps (phosphatidyl-myo-inositol capped LAM [PILAM]) (14), whereas virulent strains of mycobacteria, such as *Mycobacterium bovis* and *M. tuberculosis*, are capped extensively with α-(1→2)–linked Man*p* monosaccharide, disaccharide, and trisaccharide units (mannose-capped LAM [ManLAM]) (15, 16). It has been estimated that 40–70% of the nonreducing termini of LAM from pathogenic strains of *M. tuberculosis* are Man*p* capped (14), and analysis of the relative abundance of the different cap motifs for a virulent clinical strain showed that the dimannosyl unit was the major capping motif (∼80%), with lesser concentrations of monomannosyl and trimannosyl motifs (10–13%) (17). *M. tuberculosis* ManLAM contains an additional cap modification, 5-deoxy-5-methylthio-xylofuranose (MTX), attached to a terminal Man*p* by an α-(1→4) linkage (18), that is present in low abundance (19, 20).

Soluble LAM secreted from bacteria and infected cells is an important immunodiagnostic target for *M. tuberculosis* infection and activation (21–26). A commercial lateral flow assay that uses polyclonal Abs raised against LAM to detect this Ag in the urine of TB-infected subjects (Alere Determine TB LAM Ag) has proven useful in diagnosing TB infection in HIV-coinfected patients with low CD4 counts (25, 27–30), and the use of this test allowed early detection and subsequent treatment that was associated with reduced mortality in such patients (31). However, the sensitivity of this assay was insufficient to detect TB infection in the absence of HIV-1 coinfection, and this was therefore not recommended by the World Health Organization as a general screening test for TB (32). It has been proposed that the greater sensitivity of this assay in HIV^+^ patients may be related to TB dissemination to the kidneys, with concomitant increases in the levels of Ag present in the urine (33). This suggests that the performance of these assays could be improved by the identification of more sensitive and specific Abs that would increase the accuracy and allow a more widely useful point-of-care assay.

Despite its importance as an immunodiagnostic target and as an essential virulence factor (34, 35), little is known about the human humoral response toward LAM. Most available LAM-reactive mAbs have been derived from mice immunized with *Mycobacterium leprae* (36) or with LAM purified from *M. tuberculosis* (15). Of these, a detailed understanding of the binding specificity is known for only one, CS-35 (37–40). Although there is a recent description of a phage-derived mAb isolated from a nonimmune human Ig library that is specific for ManLAM (41), there have been no descriptions of any natural human mAbs against LAM or of any LAM-specific Ab induced in response to infection.

In recent years, conditions for culturing human memory B cells in vitro, coupled with efficient methods for molecularly cloning Ig genes from single cells, have been used to isolate novel mAbs that possess remarkable neutralization breadth and potency against HIV-1 and other viruses (42, 43). We have applied similar techniques to isolate several LAM-specific human mAbs from patients with recent diagnoses of activated TB. In this article, we describe two such mAbs and characterize their epitope specificities, along with those of a number of previously described mouse mAbs and four mAbs isolated from various Ab phage display libraries. These studies demonstrate that the human mAbs possess unique characteristics and binding specificities compared with those of previously described mAbs, and provide new information regarding the diversity and distribution of epitopes in LAM that highlights the complexity of the antigenic structure of this target.

## Materials and Methods

### Isolation of LAM-specific human mAbs

Two novel human mAbs, A194-01 and P30B9, were isolated by screening in vitro cultures of fractionated memory B cells from recently diagnosed TB subjects for ManLAM-reactive Abs. Both patients were diagnosed with active pulmonary TB, and were within the first 2 mo of antibiotic treatment. Ab H and L chains were molecularly cloned from memory B cells cultured in vitro, using modifications of previously described methods (44), and inserted into standard H and L chain expression vectors.

Memory B cells were enriched by a combination of negative selection with magnetic beads containing Abs against cell surface markers CD2, CD3, CD14, CD16, CD36, CD43, CD56, CD66b, and glycophorin A and positive selection with magnetic beads coupled to Ab against the cell surface marker CD27, a marker for memory B cells that is also expressed in low levels on plasma cells. For A194-01, the cells were cultured in vitro in 96-well culture plates at an initial concentration of ∼600 memory B cells, mixed with 15,000 MS40L-low feeder cells, in 200 μl per well. The culture medium contained the TLR9 agonist type B CpG oligonucleotide ODN 2006 (1 μM), combined with various cytokines: IL-2 (10 ng/ml), IL-4 (2 ng/ml), IL-10 (100 ng/ml), and IL-21 (100 ng/ml). MS40L cells were derived from murine stromal MS5 cells transduced with a virus expressing human CD40L (45).

After 2 wk in culture, the plates were screened by ELISA against ManLAM. In the initial assay, the total concentration of secreted Ab was estimated to be <5 μg/ml. Assuming the presence of 600 distinct Ab-producing clones in each well, this corresponds to an average IgG concentration < 10 ng/ml per B cell clone. Because of this low concentration, this method was biased toward Abs with relatively high affinities for the target Ags. Wells that gave positive signals in the first screen were recloned at dilutions calculated to give one to five cells per well and rescreened for ManLAM reactivity. The V_H_ and V_L_ regions were then cloned from multiple positive wells, inserted into H chain (IgG1) and L chain (κ) expression vectors and sequenced. All combinations of H and L chains were transfected into 293T cells, and one reactive H and L chain combination was selected for reactivity against LAM by ELISA.

Human mAb P30B9 was isolated from memory B cells obtained from patient TB314 by a modification of the method described above. The isolated memory B cells were cultured for 14 d by plating at 400 cells per well on monolayers of MS40L cells grown in 96-well plates, as described above, and cell supernatants were screened by ELISA for binding to H37Rv ManLAM with a secondary goat anti-human IgG, IgA, IgM, κ-chain reagent. After expansion, cells from a ManLAM-reactive well were transduced, as previously described (46, 47), with retroviruses expressing BCL-6 and Bcl-x_L_, two genes that have been reported to stabilize memory B cells for long-term replication. The cells were cloned out at limiting dilutions, and positive wells containing single clones were used to clone out V_H_ and V_L_ gene sequences. Initially, no activity was obtained when expressed in a standard IgG H chain vector. An analysis of the supernatant of the transduced culture showed that the reactive Ab possessed the IgM isotype, and when the V_H_ sequences were inserted into a human IgM constant domain vector and cotransfected with the corresponding L chain, LAM-binding Ab activity was detected.

Abs were expressed by transfecting H and L chain vectors in Expi293 cells in serum-free media and purified from the supernatant media by affinity chromatography on either protein A beads (for IgG) or protein L beads (for IgM), and eluted with low pH buffer. The purified Abs were concentrated and characterized by SDS-PAGE for size and purity.

### Murine mAbs

Hybridomas producing LAM-specific murine mAbs 906.7, CS-35, and CS-40 were recloned to homogeneity, and Abs from these cells and from cells producing 906.41, 908.1, and 922.5 mAbs were purified by protein A chromatography. The 900 series of mAbs (36, 48) and CS-35 (15, 49) were derived from mice immunized with extracts of *M. leprae*, whereas CS-40 was isolated after immunization with ManLAM from the Erdman strain of *M. tuberculosis* (15). The latter two Abs were produced in low yields and were difficult to purify because of solubility problems, so the variable H and L chain sequences were cloned from these hybridomas by amplification of hybridoma cDNA using a SMARTer RACE 5′/3′ Kit (catalog number 634858; TaKaRa) with a 5′ RACE universal primer and mouse C region primers, as described by the manufacturer, and expressed as chimeric mAbs with the human IgG1 Fc domain. Purified mouse mAbs FIND24, FIND29, and FIND170 (50) were isolated at the Karolinska Institute and provided by T. Broger (Foundation for Innovative New Diagnostics). These Abs gave indistinguishable results in multiple assays (Supplemental Fig. 1), so mixtures of these Abs (referred to as FIND mAbs) were used for some experiments.

### Phage-derived mAbs

Several previously described recombinant mAbs were prepared and included in these studies. My2F12 is a ManLAM-specific Ab isolated from a human nonimmune Fab-Ab phage display library by multiple rounds of positive selection with ManLAM combined with negative depletion on *M. smegmatis* PILAM (41, 51). MoAb1, MoAb2, and MoAb3 are recombinant Abs isolated at Otsuka Pharmaceutical by phage display of scFv libraries generated from rabbits (MoAb1 and MoAb3) and chickens (MoAb2) immunized with bacillus Calmette-Guérin and panned against ManLAM (52). These mAbs were expressed by synthesizing the V region sequences as gBlocks (Integrated DNA Technologies), inserting them into standard H chains (IgG1) and L chains (κ), and transfecting the paired H and L chain plasmids into Expi293 cells, as described by the manufacturer.

### Ags

ManLAM purified from *M. tuberculosis* H37Rv (ManLAM; NR-14848), *M. leprae* (LepLAM; NR-19348), and *M. smegmatis* (PILAM; NR-14860) were obtained from Colorado State University through BEI Resources, National Institute of Allergy and Infectious Diseases, National Institutes of Health. Oligosaccharide fragments of LAM and other microbial glycans were synthesized as previously described (53–57), conjugated to BSA, and used to generate a microarray (Supplemental Fig. 2). A number of these structures were also available as soluble glycoconjugates, which were used to measure Ab specificity by standard ELISAs. The oligosaccharides were prepared with an amine-containing linker at the reducing end, which was used as a reactive handle for conjugation to BSA through the use of a squarate linker, as reported (58). Briefly, each amine-functionalized glycan was reacted with diethyl squarate, and the resulting monosquaramide adduct was isolated before treatment with BSA. Loading of the glycans on the resulting BSA conjugates was determined by MALDI–mass spectrometry using sinapinic acid as the matrix (57).

Microarray assay slides were incubated at 4°C overnight and blocked by treating with BLOTTO blocking solution (Thermo Fisher Scientific). Serial dilutions of Abs diluted in BLOTTO were incubated on the slides for 30 min at 37°C; after washing, slides were stained with appropriate fluorescently labeled secondary Abs at room temperature for 40 min, washed repeatedly, dried by centrifugation thoroughly, and scanned. Microarrays were scanned at 5-μm resolution with the GenePix 4000B Microarray Scanner (Molecular Devices, Sunnyvale, CA). The excited fluorescent signal was detected by a photomultiplier tube at 532 nm for Cy3 or Alexa Fluor 555. For all microarray experiments, the laser power was 100%, and the photomultiplier tube gain was 400. The fluorescent signals were analyzed by quantifying the pixel density (intensity) of each spot using GenePix Pro Microarray Image Analysis Software 6.1. Fluorescence intensity values for each spot and its background were calculated using the software. The local background signal was automatically subtracted from the immunoassay signal of each separate spot, and the mean signal intensity of each spot was used for data analysis. Statistical analyses were performed using Microsoft Excel.

### ELISAs

LAM Ags (ManLAM, PILAM, and LepLAM) and BSA glycoconjugates were diluted in 0.05 M carbonate-bicarbonate (pH 9.6) buffer and plated at a concentration of 50 ng per well in 96-well MICROLON 200 clear round-bottom ELISA plates (catalog number 650001; Greiner Bio-One). After overnight incubation of plates at 4°C, wells were washed with PBS (pH 7.4) containing 0.05% Tween-20 (PBST) and blocked with solutions of 2% nonfat dry milk powder (Carnation) or 2% BSA (product number A3059; Sigma) in PBS buffer. Assays blocked with 2% nonfat dry milk powder had lower backgrounds but were less sensitive than those performed with BSA; overall, similar patterns were obtained. PBST-washed plates were incubated for 1 h at 37°C with appropriate dilutions of mAbs, washed with PBST, and incubated for 30 min with a 1:1000 dilution of alkaline phosphatase (AP)-conjugated goat anti-human IgG (Fcγ), IgM (Fcμ), or IgA (Fcα) chain-specific secondary Abs (Jackson ImmunoResearch). The plates were developed with p-nitrophenyl phosphate substrate in diethanolamine buffer (pH 9.8), and OD was measured at 405 nm on a UV-VIS spectrophotometer (Tecan SLT). Titers were defined, after subtracting the background OD_405_ of the BSA-coated plate, as the reciprocal dilution that produced an OD_405_ 50% of the plateau signal and were determined by exponential interpolation. Glycan- and Ab-blocking experiments were performed as described above using 2% BSA as blocking agent. For Ab-competition and LAM-capture assays, biotinylated mAbs were used as probes and detected with AP-conjugated streptavidin.

LAM-capture assays were performed using two sensitive ECL detection methods to enhance the signals. These assays used various capture Abs plated out at 20 μg/ml and biotinylated A194-01 F(ab′)_2_ (0.5 μg/ml) as the detection reagent. Signals were detected with streptavidin conjugated to AP or HRP (Jackson ImmunoResearch Labs) and developed with the appropriate substrate. AP-streptavidin was used at 1:1,000 dilution and developed with DynaLight Substrate with RapidGlow Enhancer (catalog number 4475406; Thermo Fisher Scientific) diluted 1:10 in DEA buffer (pH 9.8), whereas HRP-streptavidin was used at a 1:15,000 dilution and developed with SuperSignal ELISA Femto Substrate (catalog number 37075; Thermo Fisher Scientific). The AP format gave stronger signals, whereas the HRP format gave lower background signals and, ultimately, better signal/noise ratios. Assays were run in white LumiNunc 96-Well Plates (catalog number 437796; Thermo Fisher Scientific) with 2% BSA blocking agent. The AP plates were incubated after addition of substrate for 6–10 min at room temperature in the dark, whereas the HRP plates were read after incubation for 1 min in the dark. For both assays, relative light units were measured using a HARTA MicroLumi L2 Luminometer.

### Human subjects

Human urine samples were obtained from a panel of HIV-1^−^ TB^+^ patients, with at least one smear test positive for acid-fast bacilli, identified in pulmonary clinics at the Texas–Mexico border and from a healthy volunteer. All of the patients were on anti-TB drugs for periods of time ranging from several days to 5 wk. Human mAbs were isolated from patients with active TB disease enrolled at the Lattimore Clinic at the Global Tuberculosis Institute. Active TB disease was defined by culture-proven TB disease or a diagnosis of clinical TB. Informed written consent was obtained from all participants, and the study was approved by the Rutgers University, University of Texas Health Science Center at Houston, and Secretaria de Salud de Tamaulipas Institutional Review Boards.

## Results

### Characterization of the binding properties of a panel of LAM-specific mAbs

The Ag and epitope specificities of novel human mAbs A194-01 and P30B9 were compared with those of a number of previously described murine and recombinant phage-derived LAM-specific mAbs by ELISA against LAM from three mycobacterial species: *M. tuberculosis* (ManLAM), *M. smegmatis* (PILAM), and *M. leprae* (LepLAM). Because human and mouse mAbs of various isotypes were tested, this required the use of different secondary Abs to detect binding. To control for the different reactivities of these reagents, the secondary Abs were titrated, and dilutions and time points were selected that gave similar OD_405_ values against the corresponding primary Abs captured on ELISA plates. Nonetheless, these binding curves should be considered semiquantitative, and the shape of the curves, rather than the absolute ODs, were most informative.

**FIGURE 1.**
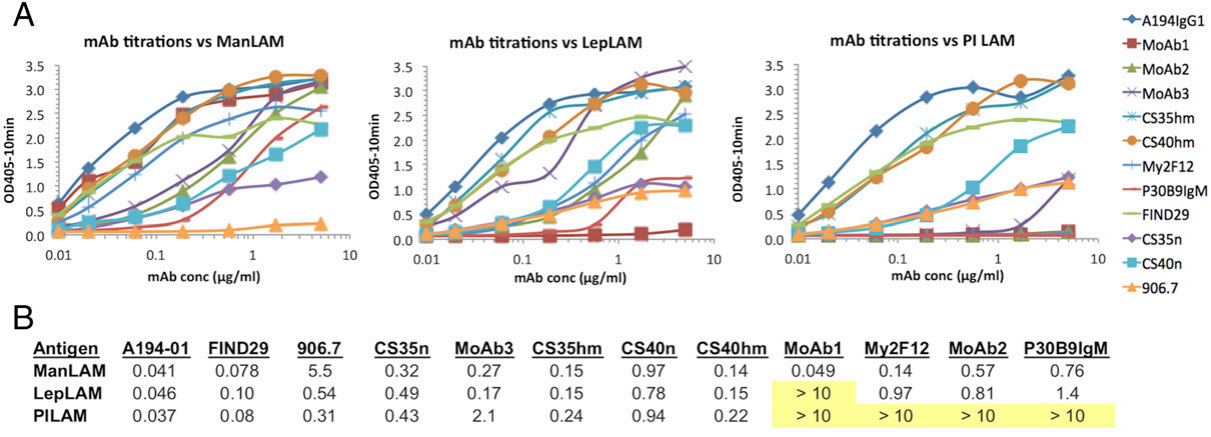
(**A**) Binding titration of mAbs to purified LAMs isolated from *M. tuberculosis* (ManLAM), *M. leprae* (LepLAM), and *M. smegmatis* (PILAM). After coating the Ags (20 μg/ml) on ELISA plates, the plates were blocked by incubation with a solution of 1% BSA and titered against a panel of 10 mAbs reactive with LAM carbohydrate structures. Ab binding was detected with the appropriate AP-labeled secondary Ab. (**B**) Tabulation of the 50% maximum binding concentration (μg/ml) of each mAb for each of the Ags. Nonreactive combinations are highlighted in yellow.

Several distinct reactivity patterns were seen for this panel of Abs. Four of the mAbs (human A194-01 and mouse mAbs FIND29, CS-35, and CS-40) were broadly cross-reactive with all three forms of LAM, with A194-01 giving the strongest activity for all three Ags (Fig. 1). Results for CS-35 and CS-40 are reported for recombinant forms of these Abs containing the human IgG1 H chain constant domains, but similar results were seen for the natural mouse Abs. The cross-reactivity of CS-40 was unexpected, because this mAb was previously reported to be specific for ManLAM (15). Another group of three mAbs (human Ab P30B9 and two phage-derived mAbs, My2F12 and MoAb2) was reactive with ManLAM and LepLAM but did not recognize PILAM, whereas a third phage-derived mAb, MoAb3, reacted preferentially with ManLAM and LepLAM and only weakly with PILAM. The highest specificity for ManLAM was seen for MoAb1, which was the only mAb that did not recognize PILAM or LepLAM. A representative of a group of mAbs isolated from mice immunized with *M. leprae*, 906.7, recognized PILAM and LepLAM with similar efficiencies but reacted only weakly with *M. tuberculosis* ManLAM.

### Determination of the epitope specificities of anti-LAM mAbs

**FIGURE 2.**
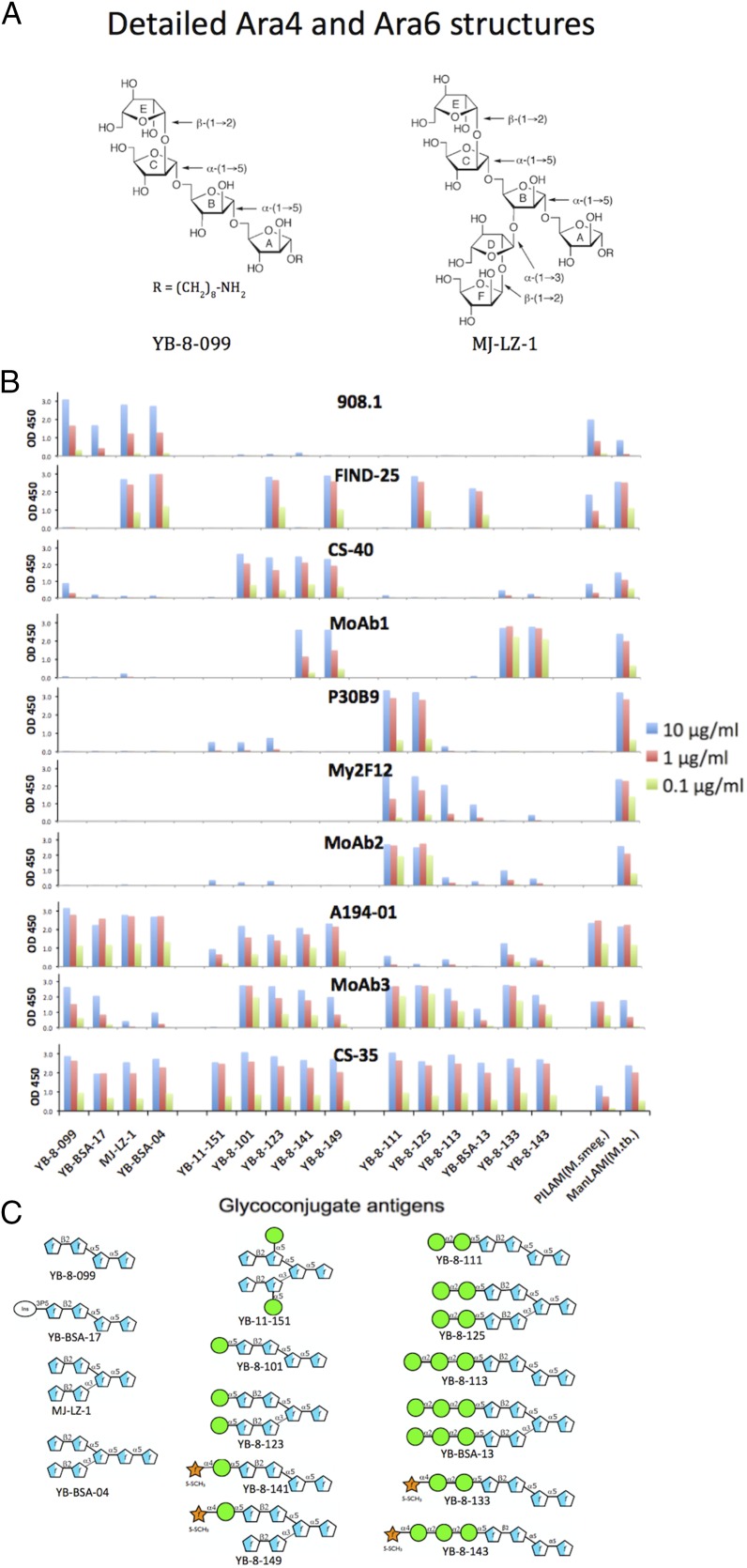
Analysis of epitope specificity of LAM-reactive mAbs by ELISA to glycoconjugates expressing synthetic glycans corresponding to motifs present in ManLAM. (**A**) Detailed structure of glycans used in Ara4 (YB-8-099) and Ara6 (MJ-LZ-1) glycoconjugates. (**B**) ELISA reactivity pattern of 10 mAbs tested at the three concentrations indicated for binding activity against a glycoconjugate panel. (**C**) Glycan structures of additional glycoconjugates used in this assay.

**FIGURE 3.**
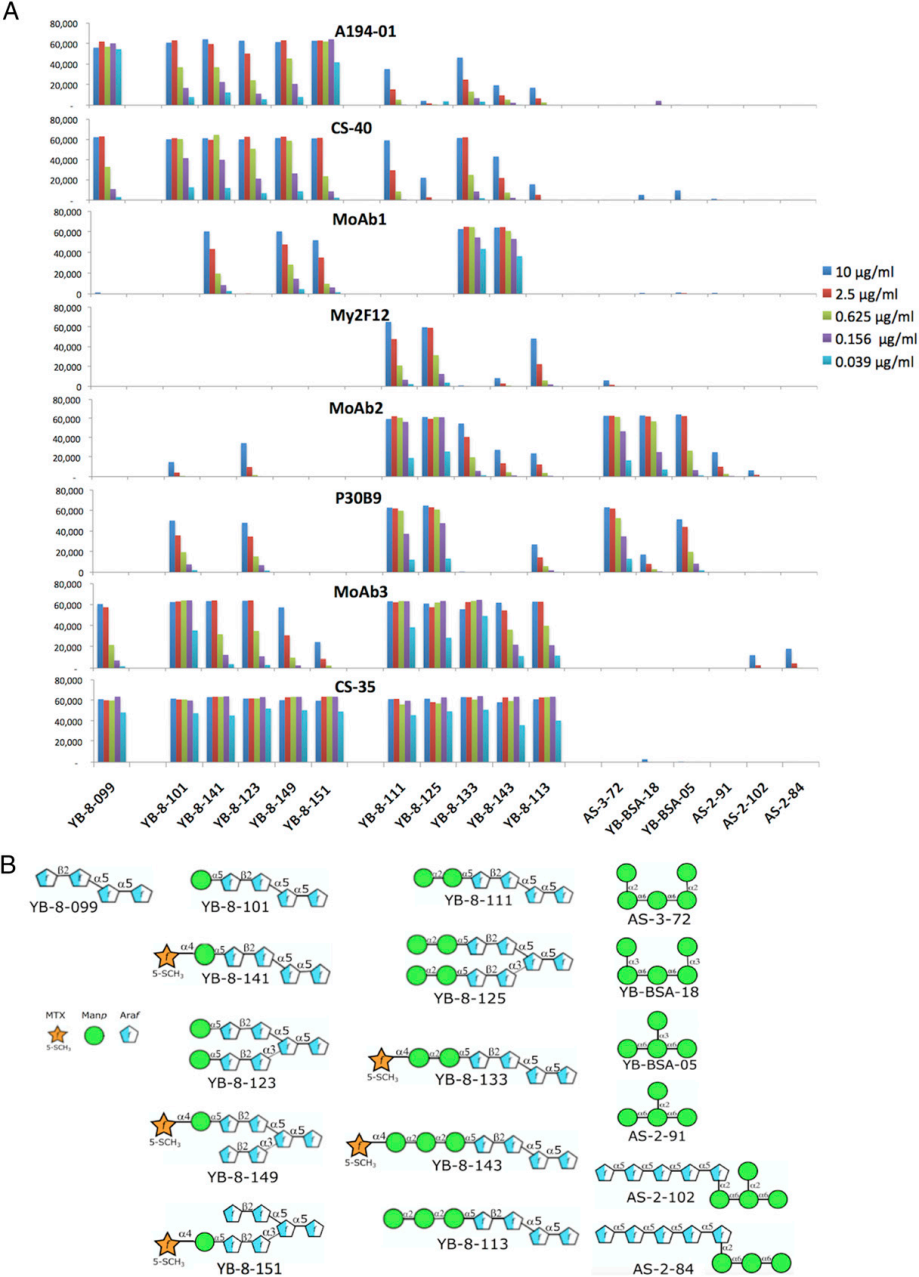
Results of a microarray analysis of the epitope specificities of mAbs dependent on mannose-capping against a subset of mannose-capped and polymannose glycan Ags shown in (**B**). (**A**) Relative Ab affinities were titrated by testing each Ab at the five concentrations indicated against the microarray, and the fluorescent signal intensities are plotted. The complete panel of structures represented in glycan microarray of microbial carbohydrates is shown in Supplemental Fig. 2, and the complete reactivity pattern is shown in Supplemental Fig. 3.

The variation in specificities of the mAb panel for LAMs from different mycobacterial species suggested that these Abs recognized distinct molecular targets in LAM. To define the epitopes recognized by these mAbs, their reactivities were tested by ELISA against a panel of BSA glycoconjugates containing synthetic carbohydrate structures that represented the basic structural motifs known to be present in various mycobacterial LAMs (Fig. 2), as well as by a microarray assay (Fig. 3, Supplemental Fig. 3) using slides containing glycoconjugates of 61 mycobacterial glycan structures (Supplemental Fig. 2) (53). The glycans in the ELISA panel consisted of poly-arabinose Ara4 and Ara6 structures, uncapped and capped with mono-, di-, and tri-Man*p* oligosaccharides, including mannose-capped structures that were further modified by attachment of an MTX residue, a modification present in *M. tuberculosis* LAM (19, 20, 55, 28). In addition to the LAM-related structures used in the ELISAs, the microarray contained a number of Ara-free polymannose structures, and structures related to other mycobacterial cell wall glycans (phenolic glycolipids, α-glucans, glycopeptidolipids, and lipo-oligosaccharides) (59).

Seven major reactivity patterns were obtained for these Abs. Mouse mAb 908.1 gave the most restricted pattern, reacting only with uncapped Ara4 and Ara6 structures and with a phospho-myo-inositol–capped Ara4 structure. Identical patterns were obtained for other members of the 900 series of Abs (Supplemental Fig. 1), suggesting that these Abs were clonally related. This limited reactivity correlated with the preferential reactivity of 906.7 with PILAM and LepLAM, which contains no or limited Man*p* capping, and weaker reactivity with ManLAM, in which Man*p*-capped structures are the dominant forms present. In contrast to this pattern, FIND25 had no activity against any of the Ara4 structures, but recognized capped and uncapped forms of Ara6 (with the exception of YB-11-151, in which the penultimate Ara*f* residues at the nonreducing ends were mannosylated). This pattern was consistent with the broad reactivity of this mAb for all three forms of LAM (Fig. 1). Identical patterns were also obtained for three additional Abs related to FIND25 (Supplemental Fig. 1), suggesting that this group of Abs is also clonally related. The FIND Ab family was unreactive with two additional structures in which the 1→5 arm (YB-BSA-06) or the 1→3 branch (YB-BSA-08) of the Ara6 structure was extended by insertion of five additional 1→5-linked Ara*f* residues between the terminal β-(1→2)–linked Ara*f* residues and the rest of the molecule [such extended motifs have been identified in LAM from *M. smegmatis* (60)]. This indicates a strong dependence on the distance between the terminal β-(1→2)–linked Ara*f* residues on either arm of the Ara6 structure and the branching point for recognition by the FIND mAbs.

CS-40 preferentially recognized Ara4 and Ara6 structures capped with a single Man*p* residue and with their MTX derivatives, and bound weakly to the Ara4 structure (YB-8-099) and structures with di-Man*p* or tri-Man*p* caps (Figs. 2, 3). Similar patterns were obtained with the natural CS-40 (Fig. 2) and with a chimeric form containing the human IgG1 Fc domain, which possessed a stronger reactivity (Fig. 3, Supplemental Fig. 3). Although recognition by CS-40 was strongly reduced by attachment of a second α-(1→2)–linked Man*p* residue to the first mannose cap, it was not affected by attachment of the α-(1→4)–linked MTX substituent, suggesting that a free hydroxyl at C-2 of the first Man*p* residue may be required for strong recognition by CS-40. The lack of recognition of YB-11-151, which contained Man*p* residues attached via an α-(1→5) linkage to internal Ara*f* residues in Ara6, also supports a strong dependence on the nature and position of the Man*p* linkage to the poly-Ara*f* chain for CS-40 binding affinity. The preference of CS-40 for a single Man*p* cap did not correspond with its reactivity with PILAM, which is not believed to possess any mannose caps (15, 17); this cross-reactivity may be mediated by the reactivity of this mAb with the unmodified Ara4 glycan.

The human mAb, P30B9 IgM, and two of the phage-derived mAbs, My2F12 and MoAb2, preferentially recognized the dimannose-capped Ara4 and Ara6 structures (Figs. 2, 3, Supplemental Fig. 3). This is consistent with the specificity of these Abs for *M. tuberculosis* and *M. leprae* LAM and their lack of reactivity with *M. smegmatis* PILAM. These mAbs differed in their fine specificities. P30B9 also reacted weakly with the mono-Man*p*–substituted Ara4 and Ara6 structures, whereas My2F12 reacted weakly with the trimannose-substituted structures, and MoAb2 weakly recognized both the mono- and trisubstituted structures. The reactivities of all three of these Abs for the mannosylated Ags was strongly or completely inhibited by addition of MTX (compare reactivity with YB-8-111 to YB-8-133), indicating that these preferentially recognized the unmodified dimannose glycan.

The microarray analysis further showed that MoAb2 and P30B9 (but not My2F12) reacted strongly with several Man*p*-containing glycoconjugates that did not contain any Ara*f* sugars (Fig. 3, Supplemental Fig. 3). These epitopes shared a common α-(1→6)–linked trimannose structure, with additional Man*p* residues linked by α-(1→2) or α-(1→3) bonds to the end or middle Man*p* of this structure and, thus, resembled the mannan backbone rather than the capping structures. The strongest reactivity for both Abs was with AS-3-72, a pentasaccharide that contained α-Man*p*-(1→2)–α-Man*p* linkages at both ends of the structure; such disaccharide motifs are present both in the mannan domain and in the dimannose caps. MoAb2 also reacted fairly strongly with YB-BSA-18, a pentasaccharide structure that contained α-Man*p*-(1→3)–α-Man*p* linkages at both ends of the molecule. Both mAbs also recognized YB-BSA-05, a tetrasaccharide that contained an α-Man*p*-(1→3) linkage at the middle Man*p* of the common trimannose structure, but bound very weakly (for MoAb2) or not at all (for P30B9) to AS-3-91, a related structure that contained an α-Man*p*-(1→2) linkage at this position. The preferential binding of these mAbs to YB-BSA-05 over AS-3-91 was surprising, because the α-Man*p*-(1→3)-α-Man*p* linkage has not been reported to be present in ManLAM. Two additional polymannose structures containing an α-(1→5)–linked Ara*f* pentamer joined by an α-(1→2) linkage to the AS-3-91 tetramannose structure (AS-3-102) or to an α-Man*p*-(1→6) trisaccharide (AS-3-84) were not recognized by any of these mAbs. Although the precise structural requirements of these epitopes are not fully defined by these results, they clearly indicate that the latter two mAbs are dependent solely on the Man*p*-containing component, and do not require any of the adjacent Ara*f* residues for recognition.

In striking contrast to these patterns, MoAb1 was uniquely specific for Man*p*-capped structures that were further substituted with an α-(1→4)–linked MTX residue. This mAb possessed the greatest reactivity with the modified dimannose (YB-8-133)– and trimannose (YB-8-143)–capped structures and weaker reactivity with the MTX-substituted Ara4 and Ara6 monomannose structures (YB-8-141 and YB-8-149). MoAb1 recognized structures in which the MTX–mannose motif was present on either the α-(1→5)–linked (YB-8-149) or α-(1→3)–linked (YB-8-151) arm of Ara6 (Fig. 3, Supplemental Fig. 3), suggesting that the complete poly-Ara*f* structure may not be critical for recognition. The MTX substitution has been identified in all *M. tuberculosis* isolates analyzed to date, and it has been reported to occur at the level of approximately one molecule per entire molecule of ManLAM (18–20, 61, 62). This structure has not been reported in *M. leprae* or *M. smegmatis*, consistent with the lack of reactivity of MoAb1 with LAM from these two species (Fig. 1).

The broadest reactivity against the glycoconjugates was seen for mouse mAb CS-35, which recognized all Ara4 and Ara6 structures with similar affinities, consistent with the broad recognition by this mAb of all three forms of LAM (Fig. 1). CS-35 reactivity appeared to be completely insensitive to the presence or absence of capping, consistent with the previously reported specificity of CS-35 for the Ara4 [β-Ara*f*-(1→2)–α-Ara*f*-(1→5)–α-Ara*f*-(1→5)–α-Ara*f*] motif present in all reactive structures (37). This binding pattern is consistent with the crystal structures of CS-35 Fab in complex with Ara4 and Ara6 (39), which showed that the nonreducing ends in Ara4 and Ara6 project away from the Ab surface, explaining why attachment of capping residues at these positions does not interfere with recognition by this mAb.

mAbs A194-01 and MoAb3 also recognized various Ara4 and Ara6 motifs but differed in their relative preferences for capped versus uncapped structures (Figs. 2, 3). A194-01 reacted strongly with the uncapped Ara4 and Ara6 structures, including the inositol phosphate–capped Ara4 (YB-BSA-17); somewhat less strongly with single Man*p*–capped structures, including their MTX-modified forms; and only weakly with di- and tri-Man*p*–capped structures. In contrast, MoAb3 recognized uncapped Ara4 and Ara6 structures with low affinity and reacted most strongly with Ara4-Man1 (YB-8-101), dimannose-capped Ara4 and Ara6 structures (YB-8-111 and YB-8-125), and an MTX-modified Ara4-Man2 structure (YB-8-133), with lesser reactivity with the MTX-modified mono- and trimannose-capped structures (Figs. 2, 3). These specificities were consistent with the broad cross-reactivity of A194-01 with all three species of LAM and the weak reactivity of MoAb3 with PILAM (Fig. 1). As expected from their reactivity with the uncapped poly-Ara structures, these mAbs did not recognize any of the polymannose structures.

### Fine specificity of determinants of arabinose-dependent mAbs

**FIGURE 4.**
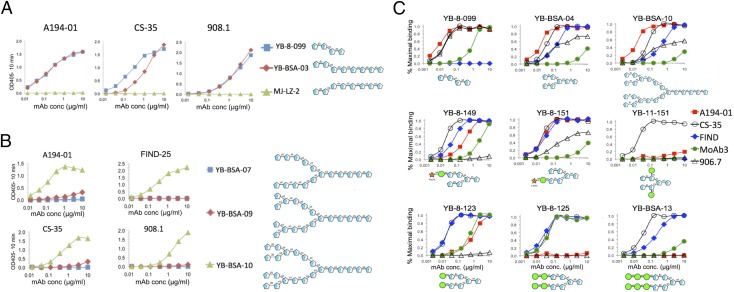
Determination of the structural requirements at the nonreducing ends of the polyarabinose chain for recognition by poly-Ara*f*–dependent mAbs by ELISA titrations. (**A**) Analysis of the importance of the Ara-α(1→5)–Ara linkage at the nonreducing sugar located at the branching point in the arabinose chain for Ab recognition by titration of the binding reactivities of the three Ara4-reactive mAbs shown. (**B**) Analysis of the requirement for the Ara-β(1→2)-Ara linkage at the terminal nonreducing position of the Ara4/Ara6 sequence by titration of the four mAbs indicated. (**C**) Analysis of the effects of capping and extensions at the nonreducing ends of the Ara6 structure on binding reactivity of the five mAbs shown. Results are presented as the percentage of maximum binding signal, rather than OD, to control for the various activities of the different secondary Abs (anti-mouse versus anti-human) used in these assays.

Further information regarding the fine epitope specificities with strong binding affinities for uncapped Ara structures was obtained from the analysis of the reactivities of these mAbs with different poly-Ara*f* variants (Fig. 4). The basic Ara4 structure consists of a β-Ara*f*-(1→2)–α-Ara*f*-(1→5)–α-Ara*f*-(1→5)–α-Ara*f*-(1→5) tetrasaccharide, whereas the Ara6 structure has an additional α-Ara*f*-(1→2)–α-Ara*f*-(1→3) disaccharide branch at the second residue (residue B in Fig. 2A). Three of the mAbs (A194-01, CS-35, and 908.1) bound strongly to structures containing uncapped Ara4 or Ara6 motifs but not to a related Ag that contained only the terminal β-Ara*f*-(1→2)–α-Ara*f*-(1→3) structure, corresponding to the lower branch of the Ara6 structure (MJ-LZ-2) (Fig. 4A). This indicates that α-(1→5) linkage between residues C and E (Fig. 2A) was required for recognition by all of these mAbs, whereas an α-(1→3) linkage [i.e., the lower branch of Ara6 (residues D and F in Fig. 2A)] was not sufficient for reactivity. This result was consistent with the x-ray structure and spectrometric analyses of CS-35 bound to synthetic LAM fragments, which showed that the key interactions of this Ab were with residues A and E in the Ara4 branch of the Ag (38, 39), and indicated that the other Ara-dependent mAbs recognized a similar structure. However, these mAbs exhibited varying sensitivities to the presence of capping motifs, suggesting that they possessed different modes of binding. In contrast to the broad reactivity of CS-35, the reactivity of the 900 series of mAbs was limited to uncapped structures, whereas A194-01 recognized some capping motifs but not others.

The requirement of the terminal β-Ara*f*-(1→2) linkage for Ab recognition was further examined by probing the reactivity of these mAbs and the Ara6-dependent FIND25 mAb to three related poly-Ara*f* structures, related to structures present in mycobacterial arabinogalactan, that differed at their nonreducing termini (Fig. 4B). All three structures contained linear α-Ara*f*-(1→5) linkages present in Ara4 and Ara6. Hexadecasaccharide YB-BSA-07 terminated with an α-Ara*f*-(1→5) residue and was completely unreactive with all of the mAbs. Octadecasaccharide YB-BSA-09 contained additional α-Ara*f* residues attached via a (1→3) linkage to the penultimate Ara*f* on both arms, corresponding to the structure present at the branching position of Ara6. This structure was weakly recognized at the higher concentrations by A194-01 and CS-35 but not by the other two mAbs. Docosanasaccharide YB-BSA-10 included the terminal β-Ara*f*-(1→2) residues at each of the branches, forming two complete Ara6 structures. This structure was recognized by all three of the mAbs, with relative binding strengths consistent with their affinities toward the natural LAM Ag. These results indicate that the terminal β-Ara*f*-(1→2)–α-Ara*f*-(1→5) disaccharide was a critical component for all of these Ara*f*-specific LAM-specific mAbs.

The differential effects of Man*p* capping on recognition of the Ara6 motif by various mAbs is further illustrated by the binding curves in Fig. 4C. As shown previously, CS-35 possessed the broadest reactivity with all of these structures, whereas 906.7 was highly specific for Ara4 and Ara6 structures containing an uncapped upper branch. A194-01 possessed the strongest reactivity with the uncapped Ara4 and Ara6 structures (top panels) and also recognized structures containing single Man*p* caps, but reacted poorly, if at all, with dimannose- and trimannose-capped structures. Man*p* attachment to the penultimate Ara*f* residues (C and D in Fig. 2A) in the Ara6 motif (YB-11-151) did not affect recognition by CS-35, but resulted in loss of reactivity for the other mAbs. The FIND mAbs recognized all of the Ara6 structures but not the Ara4 structure. MoAb3 bound to both capped and uncapped structures but preferentially recognized dimannose-capped structures.

### Characterization of epitope avidity and valency dependence by competition assays with soluble glycans

The identification by LAM-reactive mAbs of diverse epitope specificities is consistent with a complex and heterogeneous structure for LAM. In addition to the specific glycan structures required, these epitopes may be dependent on the valency of the Ab–Ag interaction and on the number and spatial orientation of binding sites accessible in LAM. Ag and Ab binding–competition assays were used to probe the relative avidities of these mAbs toward soluble glycans that define these epitopes and to define the spatial relationships of individual epitopes in ManLAM.

**FIGURE 5.**
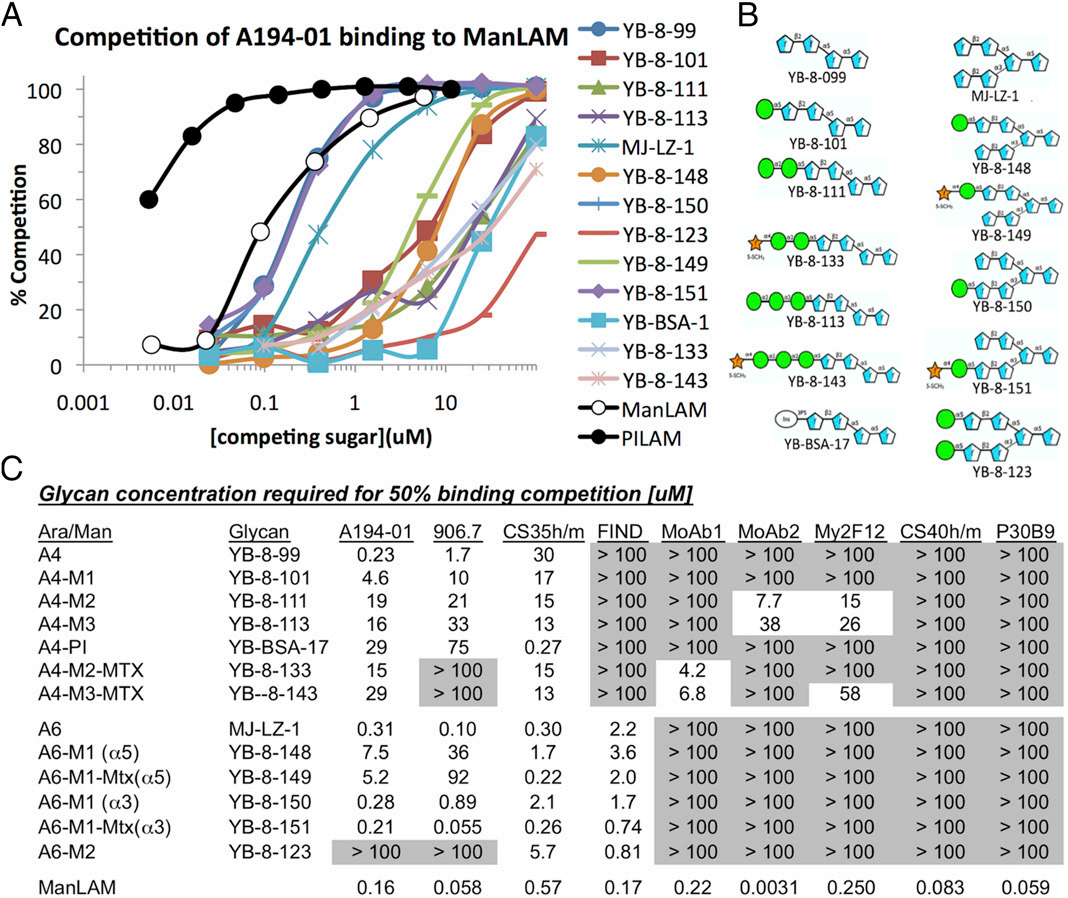
Competition of binding reactivities of nine mAbs to ManLAM by monomeric glycans and soluble ManLAM. (**A**) Competition curves for binding of A194-01 to ManLAM by soluble glycans, PILAM, and ManLAM. (**B**) Structures of competing glycans used in this assay. (**C**) Tabulation of glycan and ManLAM concentrations (micromolar) required for 50% inhibition of binding of the mAbs tested to ManLAM.

To study the effect of epitope valency on mAb recognition, the ability of soluble monomeric glycans corresponding to defined epitopes to compete for binding of mAbs to ManLAM was determined. Competition by soluble PILAM and ManLAM was used for comparison. Typical binding curves are shown for A194-01 in Fig. 5A, the available glycans used for competition are shown in Fig. 5B, and a tabulation of the glycan concentrations required to obtain 50% competition of binding of the various mAbs to ManLAM is presented in Fig. 5C.

As expected, the binding activity of all of the mAbs to immobilized ManLAM was competed by soluble ManLAM, although with different efficiencies. All of the soluble glycans competed with different efficiencies for binding of A194-01 to ManLAM in a manner consistent with the relative affinity of this Ab toward the related glycoconjugates. The most efficient competition was seen for glycans containing unmodified Ara4 (YB-8-099) or Ara6 structures with an unmodified Ara4 branch (residues A, B, C, and E in Fig. 2A), such as MJ-LZ-1, YB-8-150, and YB-8-151. Although some glycans competed on a molar basis against A194-01 as efficiently as ManLAM, these did not compete as strongly as PILAM. This presumably reflects a favored spatial arrangement of the uncapped and partially capped structures preferentially recognized by A194-01 in PILAM compared with ManLAM. Consistent with the glycoconjugate mapping data for this mAb, less efficient competition was seen for glycans in which the Ara4 branch was capped with phospho-myo-inositol or with one or more Man*p* residues, with the single Man*p*-capped Ags generally competing more strongly than the di- or tri-Man*p*–capped structures.

Three other Ara-reactive mAbs, 906.7, CS-35, and FIND, were also competed with differing affinities by soluble glycans (Fig. 5C), with sensitivities consistent with the binding affinities for the corresponding glycoconjugates. mAb 906.7 was most sensitive to uncapped Ara4 and to Ara6 structures containing an uncapped ABCE branch, and was less sensitive or unreactive to the Man*p*-capped structures. CS-35 was competed by all of the capped and uncapped glycans but was more sensitive to the Ara6 structures than to the Ara4 structures, whereas the FIND mAbs were competed similarly by all of the Ara6-based glycans but not by any of the Ara4 structures, consistent with the dependence of these mAbs on the branched Ara6 structure.

In contrast to these results, the Man*p*-dependent mAbs tested were competed inefficiently, if at all, by the monomeric glycans, including those that were recognized with high avidity when expressed as glycoconjugates. CS-40 and P30B9 were not competed at all by any of the glycans tested, whereas MoAb2 and My2F12 were competed only weakly by two and three of the dimannose-capped glycans, respectively. MoAb1 was competed only by MTX-modified dimannose- and trimannose-capped structures (YB-8-133 and YB-8-143), but not by MTX-modified monomannose structures, also consistent with the preferred recognition of the corresponding glycoconjugates by this mAb.

### Analysis of epitope topography and distribution in ManLAM by mAb cross-competition and depletion assays

**FIGURE 6.**
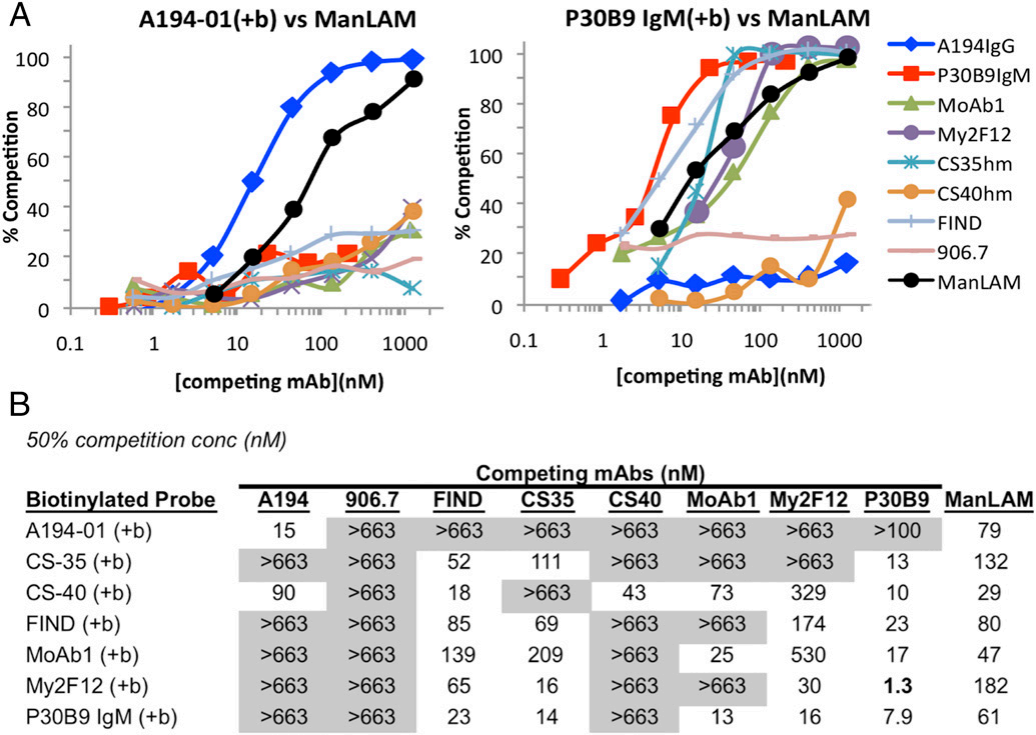
Cross-competition patterns of binding of probe mAbs to ManLAM by unlabeled mAbs and soluble ManLAM. (**A**) Competition curves for binding of biotinylated A194-01 and P30B9 to ManLAM by seven mAbs and ManLAM. (**B**) Summary of 50% blocking concentrations (nanomolar) for ManLAM and each Ab combination.

The spatial relationships between the various epitopes in ManLAM were examined by measuring the ability of excess concentrations of unlabeled Abs to compete for binding of biotinylated probe mAbs in ELISAs. Typical competition curves are shown for A194-01 and P30B9 (Fig. 6A), and the concentrations of competing Ab and soluble ManLAM required to block 50% binding of various biotinylated mAbs are tabulated (Fig. 6B). As expected, all of the mAbs were competed efficiently by their own unlabeled forms and by ManLAM, but A194-01 and P30B9 possessed very different competition patterns for the other mAbs. A194-01 was not significantly competed by any of the other mAbs, and despite its high affinity for LAM, excess unlabeled A194-01 competed only against itself and CS-40 but not against any of the other biotinylated mAbs tested. In contrast, P30B9 competed for all of the probe mAbs tested with the exception of A194-01, including broadly reactive mAbs that were not dependent on Man*p* capping, and labeled P30B9 was competed by most of the other mAbs tested but not by A194-01. Essentially all P30B9 binding activity was competed by high concentrations of MoAb1 and the FIND mAbs (Table I), suggesting a clustering of the respective epitopes. As expected, mAbs that recognized restricted epitopes, such as 906.7 and CS-40, did not compete for the more widely distributed epitopes. However, MoAb1, shown above to be dependent on MTX-substituted Man*p* caps (Fig. 3), did compete efficiently against both CS-40 and P30B9, suggesting that the majority of the structures expressing those epitopes in ManLAM either contained, or were proximal to structures containing the MTX substitution. These results were consistent with a distinct structure and restricted spatial orientation for the A194-01 epitope and suggested that the dimannose epitopes recognized by P30B9 were in spatial proximity to other Man*p*-dependent mAbs and to some of the broadly distributed epitopes.

**Table I.** Maximum level of competition of binding of biotinylated probe mAbs P30B9 and MoAb1 by excess concentrations of P30B9, MoAb1, and the FIND mAbs

Probe mAb	Competing mAb (100 or 200 μg/ml)		
	MoAb1 (%)	P30B9 (%)	FIND (%)
P30B9	98	99	100
	98	94	98
	97	100	100
MoAb1	94	47	57
	97	78	58
	100	52	ND

The percentage competition achieved at the highest concentration of competing Ab (100 or 200 μg/ml) is reported for three independent experiments.

ManLAM consists of a heterogeneous population of molecules with a considerable range in size, as determined by SDS-PAGE and mass spectrometry (16). Thus, the low level of cross-competition between A194-01 and the other mAbs raised the possibility that these epitopes were mostly segregated in distinct populations of LAM molecules. This possibility was examined by immunodepleting ManLAM over CNBr-activated Sepharose beads containing immobilized Abs and examining the residual reactivity of the unbound fraction. Absorbing 25 μg of ManLAM on beads containing 1 mg of A194-01 or P30B9 resulted in removal of >99% of molecules reactive with A194-01, P30B9, and CS-35 Abs, whereas a mock column did not remove any immunoreactive material (Supplemental Fig. 4). This result demonstrated that all of these epitopes were coexpressed on essentially all ManLAM molecules, and supported the conclusion that the lack of cross-competition between A194-01 and the other mAbs reflected a spatial segregation between the corresponding epitopes in individual molecules.

### Rational design of highly sensitive LAM-capture assays

Commercial immunodetection assays for the presence of urinary LAM in TB patients use poorly defined polyclonal Abs and possess limited sensitivity. The availability of mAbs with high affinity and well-defined epitope specificities should allow the rational development of more efficient and specific assays to detect forms of LAM present in biofluids of TB-infected subjects. To illustrate this possibility, the sensitivities of several mAb combinations were tested for detection of titrated amounts of ManLAM. The lack of competition between A194-01 and other mAbs that recognized common epitopes, such as CS-35, the FIND Abs, and P30B9, suggested that ManLAM captured with the latter mAbs would retain accessible A194-01 binding sites and that combinations of these mAbs would provide a sensitive detection assay for soluble ManLAM Ags.

**FIGURE 7.**
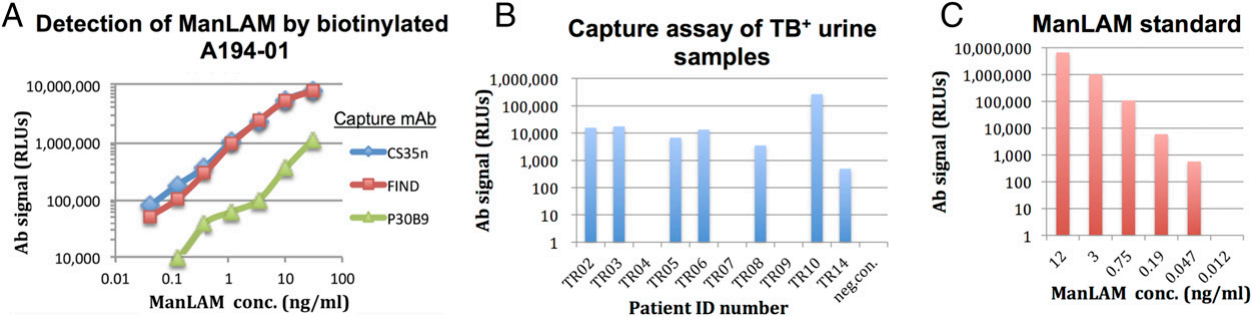
(**A**) Efficient detection of ManLAM via capture assays. ManLAM in buffer was captured in 96-well microtiter plates coated with CS-35, the FIND Abs, or P30B9 and detected with biotinylated A194-01 IgG using a sensitive ECL detection assay based on an AP-conjugated streptavidin reagent. (**B**) Demonstration of the ability to detect LAM in unprocessed urine from a panel of TB^+^/HIV-1^−^ patients, using a sensitive ECL assay based on an HRP-conjugated streptavidin reagent. (**C**) Signals obtained for titered concentrations of ManLAM diluted in buffer tested in this assay for comparison. A linear correlation was seen in this assay for the range of Ag concentrations tested and relative luminescence units (RLUs), with a lower limit <0.05 ng/ml.

LAM-capture ELISAs were performed in sensitive ECL detection formats using an AP-conjugated strepavidin reagent and CS-35, the FIND Abs, or P30B9 as the capture Abs and biotinylated A194-01 F(ab′)_2_ as the detecting reagent (Fig. 7A); the F(ab′)_2_ was chosen for the detection reagent because it gave a lower background than the intact IgG. The CS-35 and FIND capture Abs allowed the detection of concentrations of purified ManLAM < 0.1 ng/ml, whereas capture with P30B9 resulted in less sensitive detection limits (Fig. 7A). To determine whether these reagents were suitable for assaying LAM in the urine of TB patients, we tested the ability of the CS-35/A194-01 Ab pair to detect LAM in a panel of unprocessed urine samples of patients diagnosed with active TB in the absence of HIV-1 coinfection (Fig. 7B). This assay used a different ECL detection reagent based on HRP-conjugated streptavidin secondary reagent, which gave lower background signals for these samples. This assay gave a positive signal for LAM in 7 of 10 of the urine samples tested. A comparison with a standard titration curve, performed under the same conditions using purified ManLAM (Fig. 7C), suggested that the concentrations of LAM in most of these samples were quite low, in the range of 0.1–1 ng/ml.

## Discussion

This study identifies a previously unappreciated heterogeneity in the antigenic structure of ManLAM and complexity of the humoral immune response against this Ag. Current models suggest that ManLAM consists of a diverse group of structures that possess multiple poly-*Araf* branches that terminate in Ara4 and Ara6 domains that are uncapped or capped with multiple motifs, including monomannose, dimannose, and trimannose structures that are occasionally further modified by the addition of an MTX motif. The epitope-mapping studies of the mAbs characterized in this study indicate that each of these structural features contributes toward epitopes that are recognized by the LAM-specific humoral response.

These specificities could be divided into two general groups: one dependent on the poly-Ara*f* backbone and the other dependent on the presence of specific capping motifs. The first group includes mAbs that detect both Ara4 and Ara6 (A194-01, CS-35, the 900 series) and mAbs that are strictly dependent on the Ara6 motif (the FIND series). These Abs also differed in the effect of capping on their reactivity. CS-35 was completely insensitive to the presence or absence of any type of capping, and the FIND mAbs also bound well to both capped and uncapped Ara6 structures. In contrast, the 900 series of mAbs reacted only with the uncapped structures, whereas A194-01 reacted most strongly to the uncapped structures, with moderate affinity to monocapped structures, and weakly, if at all, to dimannose- and trimannose-capped structures.

The interaction of CS-35 with Ara4 and Ara6 structures has been characterized by a combination of x-ray crystallography, nuclear magnetic resonance, and other spectroscopic methods, as well as by Ab engineering and computational analyses, and shown to be mediated by multiple CH–pi interactions and hydrogen bonds between the Ab CDR regions and sites located primarily in the terminal Ara*f* residues in the Ara4 structure (residues A and E in Fig. 2A) (38–40). The epitope-mapping studies described in this study were consistent with this conclusion and further indicated that the other arabinose-reactive mAbs (A194-01, FIND, 908.1) all resembled CS-35 in their dependence on the presence of a terminal β-Araf-(1→2) residue linked to the α-Araf-(1→5) sugar present in Ara4 and in the ABCE branch of Ara6 (Figs. 4A, 4B). This appears to be an essential structure for mycobacteria, because disruption of the *aftB* gene (MSMEG_6400) responsible for the attachment of terminal β(1→2)-linked Ara*f* residues to arabinogalactan and LAM is lethal for *M. smegmatis* (63). Linkage of an α-Ara*f*-(1→5) residue to the (1→3)-linked α-Ara*f* residue corresponding to the lower branch of the Ara6 structure (residues D and F) was insufficient for reactivity of any of the mAbs, although the presence of this branch was essential for activity of the FIND mAbs. The different sensitivities of these mAbs toward various capping motifs indicated that they possess different modes of recognition, which could be further explored by x-ray crystallization studies of these Abs with appropriate soluble glycans.

The second group of mAbs included a natural human Ab (P30B9), a mouse mAb (CS-40), and the four mAbs derived from various phage display libraries (My2F12, MoAb1, MoAb2, and MoAb3). These mAbs were dependent, to various extents, on Man*p* capping and possessed different sensitivities to the number of Man*p* residues attached and the presence or absence of MTX on the terminal Man*p* residue. CS-40 and MoAb3 also bound weakly to uncapped Ara4, but they differed in that CS-40 preferentially recognized structures capped with single Man*p* residues, with weaker reactivity to dimannose- and trimannose-capped structures, whereas MoAb3 bound preferentially to dimannose-capped structures, with weaker reactivity to the other capped forms (Fig. 3). Recognition by these two mAbs was not sensitive to modification of the Man*p* caps by MTX attachment. In contrast, mAbs My2F12, MoAb2, and P30B9 did not bind to any uncapped structures, and they preferentially recognized dimannose-capped structures, with varying lower levels of sensitivity to monomannose and trimannose motifs, and these reactivities were greatly reduced by MTX attachment. The microarray binding data (Fig. 3, Supplemental Fig. 3) showed that the latter two Abs also recognized Man*p*-specific epitopes independent of any Ara*f* components, although it is likely that, in the natural Ag, the Ara*f* scaffold provides the proper orientation and multivalent expression of these sites required for Ab recognition. In contrast to the other mAbs, MoAb1 was highly specific for MTX-substituted Man*p* caps, and preferentially recognized the MTX-substituted dimannose- and trimannose-capped structures, with lower affinities to monomannose structures containing the MTX modification.

The epitope specificities of these mAbs correlated with their levels of cross-reactivity with LAMs from other mycobacterial species (Fig. 1). Man*p* capping is a conserved feature in *M. tuberculosis* and other slowly growing pathogenic strains but not for rapidly growing species, such as *M. smegmatis.* The arabinan-reactive mAbs, such as A194-01, CS-35, CS-40, and the FIND mAbs, cross-reacted strongly with ManLAM, LepLAM, and PILAM, whereas 906.7 reacted more strongly with the latter two Ags and only weakly with ManLAM, consistent with its restriction to uncapped structures. Abs that were highly dependent on Man*p* capping (i.e., P30B9, My2F12, and MoAb2) reacted with ManLAM and LepLAM, which has been reported to contain a lower level of Man*p* capping, but not with PILAM. MoAb1 was highly specific for ManLAM, which is known to possess the MTX modification and was the only Ab that did not recognize PILAM or LepLAM, indicating the absence of the MTX attachment in these Ags. A recent report described a five-gene cluster dedicated to the biosynthesis of the MTX capping motif of *M. tuberculosis* LAM (64). This cluster is not present in *M. leprae*, which contains massive deletions and gene decay compared with other mycobacteria (65, 66), and the MTX motif has previously been reported to be absent in LepLAM (67). *M. smegmatis* does have all of these genes (64), so the lack of reactivity of MoAb1 with PILAM is presumably related to the absence of Man*p* capping by *M. smegmatis*, which precludes formation of this epitope.

### Sensitivities to competition by soluble glycans reflect different dependencies on multivalency

Competition by soluble glycans was seen for some mAbs but not for others; when competition occurred, it was consistent with the epitope specificities of the Abs for the corresponding glycoconjugates. Efficient competition was seen mostly for the Ara*f*-reactive mAbs, A194-01, 906.7, CS-35, and the FIND mAbs (Fig. 5C), whereas, consistent with the glycoconjugate-mapping data, the FIND mAbs were competed by all of the Ara6 glycan structures but not by any of the Ara4 structures. In striking contrast with these patterns, competition of the Man*p*-dependent mAbs by soluble glycans was very inefficient, if seen at all, even for structures that reacted strongly when presented as glycoconjugates. Natural ManLAM and the synthetic glycoconjugates have in common a multivalent display of epitopes that allows cooperative binding of both binding sites in bivalent Abs, whereas the soluble glycans allow only monovalent binding. These results argue for a stricter dependency on multivalent interactions for the Man*p*-dependent epitopes than for the Ara*f*-dependent targets.

### Spatial orientation of epitopes in ManLAM defined by Ab-binding competition studies

Having defined the basic epitopes recognized by these mAbs, Ab-competition assays provided additional insights into the distribution of these epitopes in the native ManLAM molecules (Fig. 6). Ab competition is a reflection of the average frequency and spatial distribution of the epitopes in ManLAM. The fact that different competition patterns were seen for specific mAbs was consistent with a complex multibranched structure for ManLAM and showed that efficient Ab competition required epitope proximity, possibly with competing epitopes being localized on single terminal branches.

The efficient cross-competition among CS-35, FIND, and P30B9 suggested that the majority of these epitopes coexisted on common Ara6 structures. These results were consistent with previous structural studies indicating that dimannose caps were dominant structures in ManLAM (17), and the efficient cross-competition by the FIND mAbs further suggested that these structures were present mostly on Ara6, and not Ara4, structures. This conclusion may also explain the efficient competition of binding of P30B9 to ManLAM by MoAb1 (Fig. 6, Table I), despite the fact that for the glycoconjugates, attachment of MTX required for MoAb1 reactivity blocked recognition by P30B9 (Figs. 2, 3). A possible explanation for this result is that MTX substitution occurs frequently on one branch of the fully dimannose-capped Ara6 structures, forming structures in which one branch is recognized by P30B9, and the other branch by MoAb1, and the close proximity of these binding sites allows competition to occur. These results suggested that the MTX substitution occurs more frequently than suggested or that it occurs predominantly on immunoreactive sites in ManLAM. The incomplete reciprocal effect (the maximum competition of binding of MoAb1 by FIND and P30B9 was approximately <60%) may be due the presence of the MTX substitution on Ara4 glycans (not recognized by FIND) or on structures capped with one or three Man*p* residues (not recognized by P30B9).

Particularly striking was the unique competition pattern of A194-01. Although the A194-01 epitope was present on essentially all ManLAM molecules (Supplemental Fig. 4), binding of this Ab to ManLAM was competed only by itself and not by any of the other mAbs tested. Moreover, A194-01 did not compete for binding of the other mAbs, with the exception of CS-40 (Fig. 6B). These results are consistent with the conclusion that the A194-01 epitopes occupied a distinct site on ManLAM.

### Potential utility of these reagents and information for clinical applications

Although there is considerable evidence for roles for the terminal Man*p* residues in LAM in the interaction of these molecules with key surface receptors on immune cells (68–73), as well as immunomodulation of T cells and dendritic cells in vitro (69, 72, 74, 75), there are conflicting views about the importance of Man*p* capping in determining the survival and pathogenicity of different mycobacterial strains (reviewed in Refs. 12, 76, 77). The availability of well-characterized mAbs that are specific for distinct epitopes in ManLAM, including various types of Man*p*-dependent motifs, will facilitate the exploration of the functional contributions of each of these structures. In addition, having defined the specificity of MoAb1 as requiring the MTX motif, this mAb could be a useful tool for probing how widespread this modification occurs in various mycobacterial strains, including clinical isolates, and for evaluating potential functional roles for this modification.

As reviewed above, there is a critical need for a more sensitive and affordable point-of-care assay for the detection of LAM in blood and urine of patients with active TB. This article characterizes, for the first time to our knowledge, the specificities and binding characteristics of a large number of new and previously isolated LAM-specific mAbs and provides the basis for the rational development of an improved assay. An example of this was illustrated by the ability of the CS-35/A194-01 Ab pair to detect urinary LAM in 7 of 10 urine samples from HIV-negative patients with pulmonary TB (Fig. 7B). The low levels of LAM present in these samples (<1 ng/ml) may account for the failure of standard lateral flow assays to efficiently detect LAM in patients without severe HIV-1–induced immunosuppression, and this is consistent with a recent report using a novel chemical preconcentration step that found the frequent presence of low concentrations of LAM in HIV-1^−^ patients with active TB infections (78).

The well-characterized binding specificities of the mAbs described in this article will also help to determine which LAM structures are most abundant and accessible for binding in clinical specimens. Various assay platforms have been described that possess greater sensitivities than standard ELISAs, and these can further improve the sensitivity of these assays. More extensive LAM-detection assays based on mAbs described in this article, including pretreatment methods to enhance the accessibility of LAM in patient urine and sera (79), the testing of other combinations of these mAbs against large panels of clinical samples, and the evaluation of more sensitive assay formats, are ongoing and will be reported separately.

## Supplementary Material

Data Supplement
