# Supplementary material for: Characterization of the Antigenic Heterogeneity of Lipoarabinomannan, the Major Surface Glycolipid of Mycobacterium tuberculosis, and Complexity of Antibody Specificities toward This Antigen
Source: J Immunol. 2018 Apr 2;200(9):3053–66. doi: 10.4049/jimmunol.1701673 (PMC5911930; doi:10.4049/jimmunol.1701673)
Supplement: Data Supplement [file JI_1701673.zip › JI_1701673_Supplemental_Figures_1.pdf]

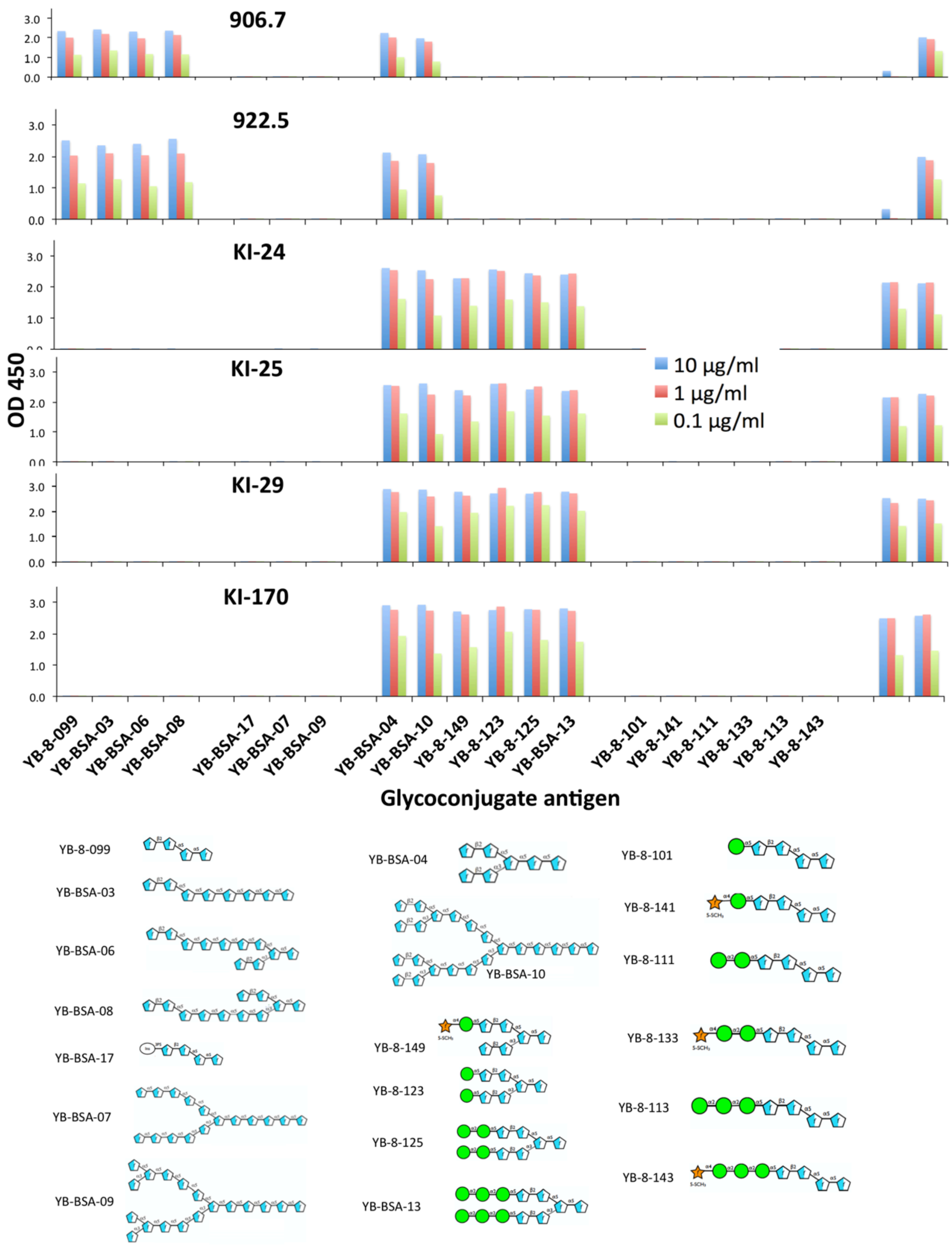

Supplementary Fig. 1. Analysis of binding patterns of additional members of the 900 and FIND series of mAbs, showing that the antibodies within each of these groups possess identical epitope specificities.

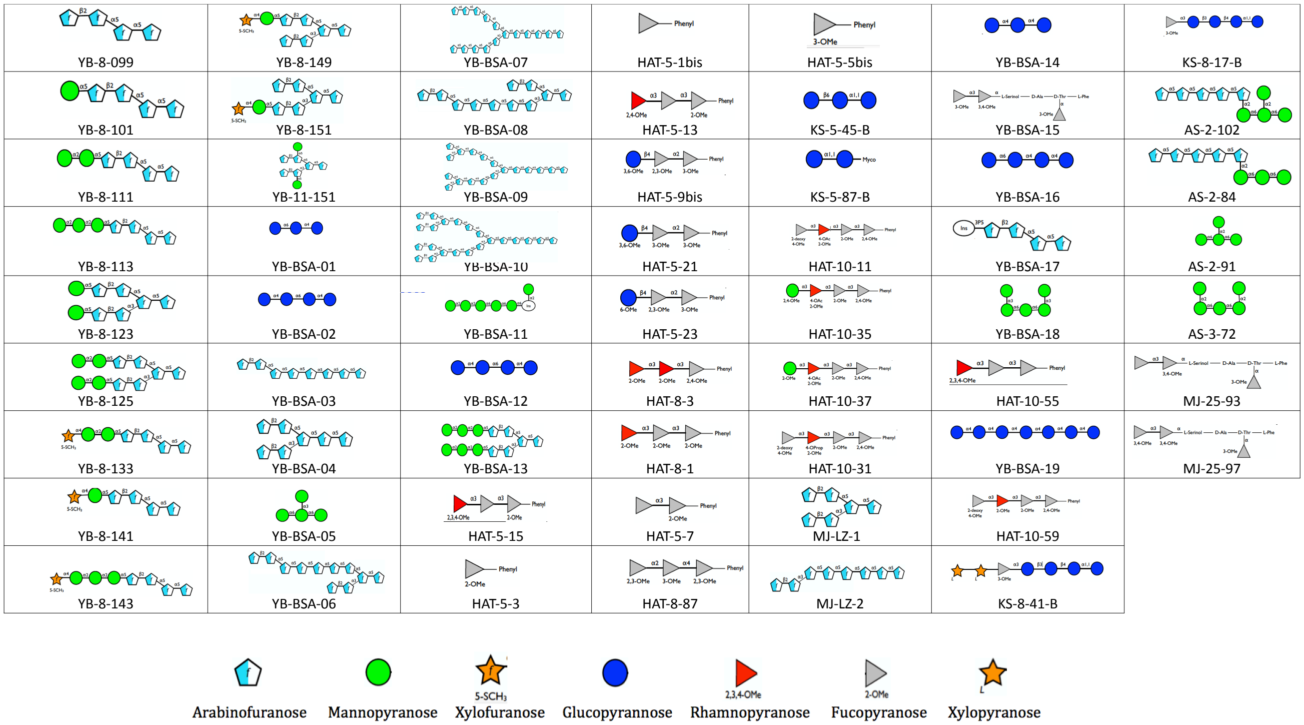

Supplementary Fig. 2. Structures of 61 glycan structures present in the microbial glycoarray used to probe the specificities of monoclonal antibodies. The key to the symbols used is shown at bottom of the figure.

		S1	S2	S3	S4	S5	S6	S7	S8	S9	S10	S11	S12	S13	S14	S15	S16	S17	S18	S19	S20	S21	S22	S23	S24	S25	S26	S27	S28	S29	S30	S31
mAb	Conc (µg/mL)	YB-8-099	YB-8-101	YB-8-111	YB-8-113	YB-8-123	YB-8-125	YB-8-133	YB-8-141	YB-8-143	YB-8-149	YB-8-151	YB-11-151	YB-BSA-01	YB-BSA-02	YB-BSA-03	YB-BSA-04	YB-BSA-05	YB-BSA-06	YB-BSA-07	YB-BSA-08	YB-BSA-09	YB-BSA-10	YB-BSA-11	YB-BSA-12	YB-BSA-13	HAT-5-15	HAT-5-3	HAT-5-1bis	HAT-5-13	HAT-5-9bis	HAT-5-21
A194-01	2.5	62,325	62,533	19,807	9,428	43,051	4,104	24,880	54,299	11,948	63,578	61,166	12,300	7	32	63,830	65,145	325	61,328	708	64,451	2,688	54,037	60	192	2,439	18	41	24	2	3	2
A194-01	0.625	61,857	39,702	6,139	3,588	21,392	1,291	9,195	33,165	5,997	49,591	61,797	8,201	4	20	64,772	64,761	80	64,913	281	64,896	1,767	59,083	53	90	898	8	44	88	15	9	2
A194-01	0.15	65,143	26,628	2,467	2,024	16,685	531	8,747	25,859	4,381	35,166	64,068	6,338	3	13	64,754	64,965	40	63,785	62	63,826	1,096	58,828	19	45	429	16	30	27	4	0	5
A194-01	0.039	60,018	12,886	661	815	10,006	111	5,513	16,320	2,420	18,623	44,529	3,165	5	7	57,004	50,529	30	56,868	39	51,699	577	44,662	17	33	308	10	23	214	13	2	0
906.7 ms	2.5	40,943	1,717	67	87	1,719	60	601	2,133	2,056	1,730	26,294	1,197	56	58	26,446	29,646	86	28,962	450	26,975	2,932	21,172	66	83	88	71	58	96	56	60	51
906.7 ms	0.625	37,999	699	46	55	957	41	164	951	85	807	21,001	556	43	47	30,907	23,483	65	26,494	450	23,013	875	18,704	52	69	60	70	50	81	50	47	42
906.7 ms	0.15	38,951	202	42	44	115	38	75	448	54	93	14,864	83	42	43	31,025	20,522	48	23,726	68	19,974	172	14,462	43	56	44	56	52	52	41	44	40
906.7 ms	0.039	30,272	62	40	42	56	37	78	76	44	52	6,797	49	48	40	21,835	10,467	39	11,854	47	11,165	80	6,089	38	38	42	40	47	48	39	38	42
FIND	2.5	6	4	-39	-73	62,390	61,790	3	7	-6	63,457	61,310	324	2	3	4	49,050	143	47	111	12	16	57,631	64	24	62,699	13	25	46	21	2	-6
FIND	0.625	5	1	1	-37	64,613	61,435	1	2	2	63,954	65,099	32	1	1	2	59,884	38	3	-1	3	1	35,381	30	1	50,250	2	1	3	30	2	-1
FIND	0.15	6	1	1	-9	64,731	63,651	2	1	2	50,200	59,049	30	1	1	2	33,284	18	25	-1	2	1	14,287	19	5	28,062	2	1	4	4	1	0
FIND	0.039	3	2	1	-1	50,979	41,270	0	1	1	23,825	36,633	0	2	1	2	13,498	13	2	0	1	1	4,638	16	1	12,805	1	1	4	7	1	0
CS35msms	2.5	58,031	59,969	52,779	51,964	55,786	50,815	58,650	55,265	47,910	52,994	50,088	51,680	9	2	33,904	50,665	10	25,751	3	48,605	143	42,794	4	5	38,632	8	3	6	1	3	43
CS35msms	0.625	57,179	58,173	51,469	47,845	55,767	55,633	56,372	51,152	40,801	49,905	46,704	56,991	3	-1	14,551	42,619	7	11,296	-8	39,121	56	32,736	2	5	34,148	8	2	3	4	5	-4
CS35msms	0.15	39,860	38,366	31,928	29,174	37,437	39,265	39,780	32,470	24,692	34,563	35,716	39,125	6	-8	2,855	26,922	3	855	-2	29,690	22	14,886	2	14	27,923	-11	3	4	1	4	-3
CS35msms	0.039	18,905	18,905	15,532	12,835	13,361	20,423	20,026	16,836	11,952	17,097	17,402	19,831	3	0	246	11,970	4	251	0	16,282	11	3,268	2	10	13,982	1	2	4	0	2	1
CS35 hums	2.5	60,203	60,929	61,575	62,961	62,053	58,421	63,178	63,609	63,010	63,119	63,654	61,525	764	-56	62,996	63,315	135	64,239	310	64,540	8,611	62,662	63	60	63,838	2	63	26	61	38	-154
CS35 hums	0.625	60,003	60,932	56,173	63,265	61,970	57,162	60,850	63,526	59,483	63,546	63,875	61,741	100	158	55,553	63,045	29	64,945	105	63,475	2,782	64,043	11	22	61,788	9	55	7	2	17	209
CS35 hums	0.15	63,733	60,011	59,779	63,694	63,374	63,051	64,289	63,973	63,648	63,543	63,690	63,747	-17	-47	46,952	62,564	58	47,318	-5	64,903	505	50,865	25	25	63,721	-10	10	-5	12	18	-13
CS35 hums	0.039	48,408	47,657	45,808	40,490	52,145	49,520	51,079	45,463	36,052	50,653	49,316	50,269	13	-19	14,597	34,052	3	14,637	1	41,216	164	16,311	3	3	35,756	1	2	4	5	7	-2
MoAb3	2.5	57,613	63,125	62,365	62,932	64,085	57,643	62,544	64,085	54,585	30,908	8,673	480	5	1	23,957	8,360	-18	15,870	1,184	18,396	3,203	6,396	4	-79	11,028	-3	-2	-1	2	-21	-3
MoAb3	0.625	21,767	64,045	63,558	39,988	35,064	62,143	63,643	31,901	36,267	9,698	2,446	8	3	1	7,226	1,856	4	5,103	87	4,891	241	453	2	2	3,819	14	1	3	1	1	0
MoAb3	0.15	7,328	64,147	63,364	21,554	10,930	63,613	64,674	12,300	21,993	2,665	141	15	5	5	1,622	131	2	328	21	361	70	106	5	1	294	1	1	8	1	2	1
MoAb3	0.039	1,854	35,628	38,627	11,647	3,177	28,623	49,421	3,940	11,243	219	35	2	-1	1	142	33	1	111	7	97	17	26	1	1	97	1	1	3	1	1	1
CS40 msms	2.5	63,129	61,398	29,592	5,312	62,753	2,800	62,212	59,707	21,984	62,845	61,740	11,715	83	314	22,490	15,716	285	15,180	937	19,498	1,305	3,120	15	750	4,378	-91	109	-235	-234	-300	-21
CS40 msms	0.625	32,969	60,497	8,604	161	50,863	165	25,023	64,705	7,411	58,805	23,657	1,891	61	70	6,199	3,403	103	3,369	171	5,277	100	273	25	166	427	-35	42	-50	-33	-63	-20
CS40 msms	0.15	10,956	41,644	625	49	21,326	63	8,588	39,986	2,307	26,429	8,699	216	27	19	452	309	29	340	61	382	32	74	9	49	137	-3	15	-4	-6	-9	5
CS40 msms	0.039	3,117	12,741	163	22	6,945	26	1,940	12,056	234	8,867	2,495	75	15	8	101	88	16	100	21	92	12	26	9	24	41	5	7	16	3	5	6
CS40 hums	2.5	61,207	60,099	25,094	4,044	62,151	3,335	61,741	63,238	18,639	60,732	63,815	10,401	110	56	24,337	12,419	882	21,165	2,072	15,675	976	3,623	199	3,624	4,339	-111	-25	-84	-49	-57	-81
CS40 hums	0.625	54,801	61,066	6,096	301	63,802	337	31,378	64,161	7,446	63,854	38,625	1,985	30	-23	6,764	3,163	107	4,438	150	3,148	88	276	46	251	271	-73	7	-22	-8	-1	-11
CS40 hums	0.15	34,062	60,455	3,270	43	58,289	48	17,061	64,619	3,971	61,365	19,175	736	18	25	3,467	1,556	45	2,040	89	2,166	62	115	34	120	170	-8	19	-4	0	2	0
CS40 hums	0.039	6,475	26,211	165	-23	14,780	8	3,095	23,476	231	20,076	3,742	64	7	-11	138	69	16	122	18	74	16	29	11	25	31	1	6	52	4	5	5
MoAb1	2.5	35	38	30	32	470	1	65,124	43,670	64,888	47,975	35,314	13	17	59	87	110	819	843	1,342	171	1,291	864	61	728	565	18	47	32	33	31	-6
MoAb1	0.625	19	6	13	2	1	-4	64,852	19,792	61,071	28,459	9,907	0	15	9	29	14	55	175	571	17	374	582	7	45	45	1	8	7	-2	0	3
MoAb1	0.15	11	14	12	6	10	2	54,826	8,601	53,362	14,741	6,357	4	23	3	39	18	27	24	196	8	152	295	4	22	19	1	8	6	2	6	1
MoAb1	0.039	10	0	5	2	-1	-2	43,748	2,916	36,728	4,772	1,726	-1	5	-1	9	3	5	9	21	1	19	26	0	3	4	9	1	3	0	-1	0
My2F12	2.5	8	0	47,861	22,498	1	59,254	310	-1	2,987	10	8	3	1	-2	12	19	5	205	589	9	525	675	1,737	64	10,447	-4	2	3	2	1	-1
My2F12	0.625	5	0	21,118	5,998	-1	31,540	28	-1	830	2	1	2	1	-1	5	2	-1	20	190	2	69	165	300	7	2,939	-1	1	3	0	0	0
My2F12	0.15	2	0	6,723	1,994	-1	12,604	7	-1	121	1	0	-1	0	-1	4	2	-1	7	26	1	21	32	39	1	1,106	-1	1	3	0	-1	1
My2F12	0.039	2	0	2,387	554	-1	3,840	1	-1	24	1	-1	-2	0	-2	4	2	-2	3	7	1	6	10	12	-1	245	-1	1	2	0	-1	0
MoAb2	2.5	15	4,240	62,684	12,592	9,969	59,917	41,235	-1	13,858	5	11	9,087	2	7	8	5	62,832	182	9	4	9	7	60	34	1,107	2	3	7	3	6	2
MoAb2	0.625	11	814	61,103	3,894	1,676	61,900	20,133	6	4,627	12	14	1,756	2	6	29	6	26,953	10	3	3	4	3	2	5	373	1	3	4	2	3	1
MoAb2	0.15	6	68	56,785	896	425	61,653	5,948	23	1,255	22	3	364	1	1	5	4	6,761	22	3	3	5	2	2	9	43	2	3	4	2	4	10
MoAb2	0.039	5	18	19,459	78	39	26,063	1,505	4	111	4	2	33	1	1	5	3	1,396	5	1	2	2	3	2	2	11	1	1	4	2	1	1
P30B9-huIgM	2.5	10	35,901	62,150	14,487	34,773	63,266	119	12	20	8	15	33,167	-1	4	7	11	44,219	15	25	5	21	27	4,937	9	1,087	-2	-1	0	-3	-1	-1
P30B9-huIgM	0.625	11	19,561	59,905	5,889	15,425	61,123	20	2	10	5	3	14,600	0	1	9	6	19,926	12	13	5	12	14	1,439	6	354	0	1	1	-1	0	0
P30B9-huIgM	0.15	12	7,647	37,450	1,865	6,943	47,823	5	1	5	5	3	5,120	0	0	9	8	8,326	17	7	4	7	10	200	6	33	0	1	0	-1	0	0
P30B9-huIgM	0.039	9	2,075	12,324	435	1,687	13,343	2	0	2	5	2	1,339	0	0	6	6	1,940	19	10	4	9	13	27	7	12	0	1	2	0	0	0

		S32	S33	S34	S35	S36	S37	S38	S39	S40	S41	S42	S43	S44	S45	S46	S47	S48	S49	S50	S51	S52	S53	S54	S55	S56	S57	S58	S59	S60	S61	
mAb	Conc (µg/mL)	HAT-5-23	HAT-8-3	HAT-8-1	HAT-5-7	HAT-8-87	HAT-5-5bis	KS-5-45-B	KS-5-87-B	HAT-10-11	HAT-10-35	HAT-10-37	HAT-10-31	MJ-LZ-1	MJ-LZ-2	YB-BSA-14	YB-BSA-15	YB-BSA-16	YB-BSA-17	YB-BSA-18	HAT-10-55	YB-BSA-19	HAT-10-59	KS-8-41-B	KS-8-17-B	AS-2-102	AS-2-84	AS-2-91	AS-3-72	MJ-25-93	MJ-25-97	BSA
A194-01	2.5	6	13	10	7	2	6	1	4	12	3	0	16	24,441	5,329	9	6	2	60,529	51	4	18	3	-3	1	37	12	10	1	20	5	41
A194-01	0.625	7	19	22	12	3	5	4	12	14	11	2	15	19,722	1,802	12	12	4	57,009	25	9	16	7	2,101	-2	25	17	17	11	71	1	38
A194-01	0.15	5	5	9	0	-1	1	-1	1	6	-2	-2	1	16,231	635	2	-1	-1	51,659	9	1	4	-1	-3	57	16	4	2	-1	0	4	41
A194-01	0.039	2	13	12	3	0	-1	6	10	8	1	-2	0	8,396	46	3	1	-3	32,945	1	3	2	-1	-2	5	8	4	2	1	1	2	41
906.7 ms	2.5	54	56	510	55	56	51	51	51	60	53	54	53	2,999	96	57	54	57	15,666	54	56	54	53	53	49	4,012	6,385	57	52	56	49	50
906.7 ms	0.625	52	55	74	43	43	42	44	43	49	42	42	43	1,819	53	46	42	41	10,937	42	42	44	46	40	42	865	1,464	44	41	42	41	40
906.7 ms	0.15	46	47	50	41	42	40	42	44	46	40	40	40	819	46	43	40	40	6,567	41	43	42	39	40	39	59	85	43	40	42	40	40
906.7 ms	0.039	39	44	43	39	38	42	38	42	43	39	38	40	113	42	41	39	37	2,109	38	42	40	39	37	40	42	48	41	39	40	41	38
FIND	2.5	53	8	11	10	2	5	545	6	4	3	4	-18	60,729	4	1	42	3	-18	-48	2	1	2	3	-3	2	6	2	2	7	-1	4
FIND	0.625	3	2	1	18	2	0	12	2	2	16	1	6	61,148	2	1	9	1	1	105	2	0	1	1	1	16	2	1	2	3	-2	7
FIND	0.15	0	3	1	4	1	1	4	4	2	2	2	2	40,241	2	1	4	2	-1	-24	1	0	2	5	1	2	2	1	2	6	0	0
FIND	0.039	0	1	0	3	1	2	3	2	1	2	1	2	23,416	1	2	3	1	1	-1	1	1	2	2	2	2	4	1	2	9	3	2
CS35msms	2.5	0	1	1	4	4	-1	-6	2	1	2	4	-25	50,520	105	2	85	3	56,618	7	1	5	3	3	7	3	3	3	3	5	6	1
CS35msms	0.625	0	1	2	6	3	-4	13	3	6	3	9	-13	46,459	40	4	73	10	50,233	-6	3	4	4	7	-7	1	5	4	3	11	-2	2
CS35msms	0.15	-6	2	2	3	2	1	21	3	1	1	3	-5	34,054	13	2	53	3	34,773	-1	2	3	2	2	9	3	3	2	2	2	0	1
CS35msms	0.039	-2	3	2	4	4	1	-62	2	1	1	3	-4	16,690	5	1	35	3	17,654	-14	2	1	2	3	-2	1	2	1	1	4	0	1
CS35 hums	2.5	-22	28	11	27	26	-67	2,275	6	18	18	12	-188	58,603	10,838	12	291	28	62,519	42	10	28	18	14	-97	10	32	8	9	16	-4	1
CS35 hums	0.625	19	31	12	6	6	7	-350	18	9	3	4	-195	59,474	4,993	9	212	4	60,740	17	18	15	4	2	-189	27	17	8	4	2	-19	3
CS35 hums	0.15	-1	33	3	15	8	1	-26	32	4	6	6	-14	61,189	2,321	6	186	6	63,659	-370	27	16	5	3	-84	-61	32	6	8	5	-22	2
CS35 hums	0.039	1	3	3	8	5	19	13	3	5	5	4	4	48,137	277	6	81	4	47,485	-173	4	7	9	3	-103	12	8	8	10	5	-33	-5
MoAb3	2.5	-3	-1	-2	-1	-6	-3	-5	-1	-2	-3	-2	-9	17,957	15	4	-2	37	39,879	-9	-2	1	-3	0	-15	2,777	4,615	-2	-1	2	-5	5
MoAb3	0.625	3	2	2	3	3	0	4	2	0	1	2	0	6,137	9	3	3	11	15,484	-1	5	1	1	1	-4	196	335	3	3	3	0	1
MoAb3	0.15	1	1	1	2	2	1	2	2	1	1	1	1	2,022	4	1	1	3	4,312	0	1	1	0	1	0	46	74	1	1	6	1	1
MoAb3	0.039	1	2	2	3	1	1	2	2	1	1	1	0	155	3	1	1	12	354	1	1	2	1	1	1	14	24	1	1	3	0	0
CS40 msms	2.5	-18	10	-54	-362	-368	111	-21	-25	-115	-311	-258	-15	18,703	-10	450	-231	353	31,002	462	-205	100	-265	-446	-297	-48	104	426	-215	-246	-144	-225
CS40 msms	0.625	-11	-3	-16	-69	-68	39	-49	-7	-28	-58	-51	-14	5,933	0	108	-33	70	9,422	143	-36	53	-38	-83	-83	7	79	134	-36	-42	-43	-57
CS40 msms	0.15	3	6	2	-15	-15	10	-7	5	-3	-12	-17	-2	333	7	44	-4	27	779	43	-3	19	-7	-19	-12	10	27	55	-1	-9	-7	-10
CS40 msms	0.039	6	5	11	3	4	11	5	10	4	2	9	6	86	5	17	4	240	179	16	5	12	3	-1	3	8	13	22	5	6	4	2
CS40 hums	2.5	-50	-65	-72	-120	-100	32	-104	-25	-49	-119	-149	-79	13,335	-19	1,823	15	499	34,534	343	-103	393	-68	-217	-171	-28	97	788	-66	-129	-109	-114
CS40 hums	0.625	-59	-18	-13	-26	-11	5	-49	-15	-8	-24	-27	-12	3,413	-2	97	-16	95	9,551	106	-25	33	-31	-53	-45	6	21	103	-23	-36	-15	-29
CS40 hums	0.15	0	5	1	-5	-4	17	-9	3	1	-8	-9	-2	2,046	6	53	1	47	4,915	65	-7	26	-3	-13	-19	13	32	72	2	5	-4	-11
CS40 hums	0.039	5	4	5	7	7	8	4	4	4	3	3	4	64	5	12	5	20	199	9	1	7	4	3	2	8	10	14	6	6	3	1
MoAb1	2.5	28	34	32	50	30	-50	123	22	19	12	12	28	2,661	4,072	90	88	83	25	53	-3	34	30	-13	-49	0	35	66	21	53	1,217	195
MoAb1	0.625	3	7	5	4	3	4	-3	-3	19	3	-2	10	691	894	20	25	10	7	16	2	8	4	-10	-13	5	11	13	3	7	74	77
MoAb1	0.15	-2	5	3	5	2	0	-4	-1	8	4	4	3	146	289	8	13	8	2	2	0	3	2	1	-5	2	6	7	2	6	60	50
MoAb1	0.039	-1	2	3	1	2	0	-3	0	4	0	4	-1	21	29	5	2	-1	-1	-1	1	1	0	-3	-3	3	4	3	1	1	7	43
My2F12	2.5	-2	3	4	3	-1	-1	-4	-1	5	0	-2	-2	154	334	3	-1	0	1	1	0	2	-1	-4	-5	1	4	5	1,648	1	0	48
My2F12	0.625	-1	2	3	1	0	0	-2	5	4	0	-1	-2	6	16	0	0	-2	0	-1	1	1	0	-1	-2	3	5	2	285	2	1	39
My2F12	0.15	-1	2	3	0	-1	0	-2	0	3	-1	-1	0	4	11	1	2	-2	-1	-1	2	1	0	-1	-1	3	5	2	37	1	1	38
My2F12	0.039	2	2	2	0	-1	0	-2	0	3	-1	-1	-1	-1	3	0	0	-2	2	-2	1	0	0	-1	-2	2	4	1	12	1	0	38
		1																														
MoAb2	2.5	4	3	3	8	6	2	12	3	4	3	5	0	30	4	14,433	5	8	8	62,456	2	12	16	37	-1	1,914	6	10,334	63,195	125	1	5
MoAb2	0.625	1	2	2	4	5	2	4	3	1	2	5	4	80	2	3,433	5	65	8	57,343	2	3	420	23	5	312	5	2,838	61,939	9	9	1
MoAb2	0.15	-1	3	1	3	3	1	3	2	4	1	7	1	5	2	716	5	12	3	25,513	1	1	16	6	0	36	4	719	47,062	8	0	3
MoAb2	0.039	-1	2	1	3	1	1	9	3	2	1	1	2	3	5	71	2	6	2	7,537	1	0	2	7	2	16	4	68	17,004	5	1	1
P30B9-huIgM	2.5	-1	-1	-2	-1	1	2	1	2	11	3	-1	-1	31	55	0	3	1	1	8,076	-3	-1	-1	-3	-3	2	0	13	62,056	1	2	1
P30B9-huIgM	0.625	0	0	0	1	0	2	0	0	0	0	0	0	9	10	1	2	1	2	3,042	0	1	0	-1	0	1	9	3	52,718	1	1	1
P30B9-huIgM	0.15	-1	0	0	21	1	2	-1	0	0	0	0	0	5	5	-1	2	2	2	855	0	1	-1	0	0	1	1	2	34,959	1	1	2
P30B9-huIgM	0.039	0	0	0	1	0	2	0	0	0	0	0	0	3	3	1	62	0	2	196	0	2	0	0	2	1	1	2	13,171	1	1	1

Supplementary Fig. 3. Binding analysis of binding of titered solutions of the mAbs to microarray of 61 microbial glycan structures and control proteins (shown in Supplementary Fig. 2). Reactivities are indicated by a heatmap, to highlight reactivity patterns

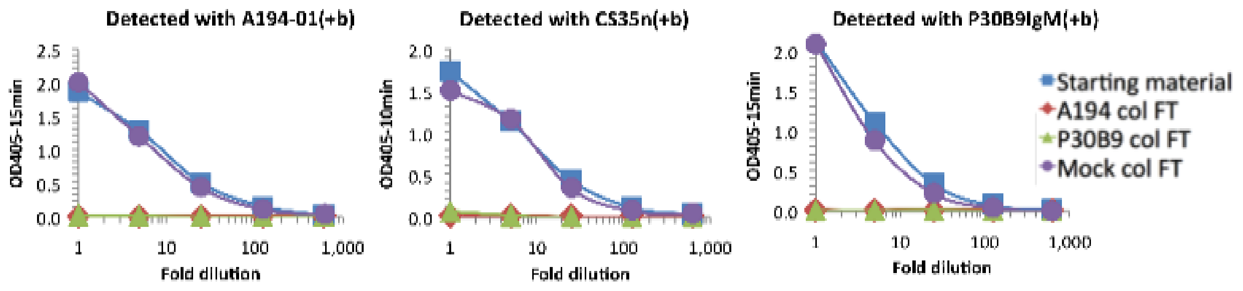

Supplemental Fig. 4. Immunoabsorption of ManLAM on mAb columns shows uniform distribution of epitopes. Analysis of immunoreactivity of ManLAM after absorption on columns containing 1 mg of immobilized A194-01 and P30B9 or a non-reactive control antibody. The reactivity of starting antigen (blue squares) was compared to that of unabsorbed residual material obtained after passage of 25 μg of ManLAM antigen over the columns. Red diamonds represent the flow-through of A194-01 column, green triangle represent the flow-through of the P30B9 column, while the purple circles represent the flow-through of the control column. All three columns removed essentially all of the immunoreactive LAM from solution, indicating that these epitopes were distributed on all molecules of the antigen.
